# Identification macrophage signatures in prostate cancer by single-cell sequencing and machine learning

**DOI:** 10.1007/s00262-024-03633-5

**Published:** 2024-02-13

**Authors:** Zhen Kang, Yu-Xuan Zhao, Ren Shun Qian Qiu, Dong-Ning Chen, Qing-Shui Zheng, Xue-Yi Xue, Ning Xu, Yong Wei

**Affiliations:** 1 Department of Urology, Urology Research Institute, The First Affiliated Hospital, Fujian Medical University, Fuzhou, 350212 China; 2 Department of Urology, National Region Medical Centre, Binhai Campus of the First Affiliated Hospital, Fujian Medical University, Fuzhou, 350212 China; 3 Fujian Key Laboratory of Precision Medicine for Cancer, The First Affiliated Hospital, Fujian Medical University, Fuzhou, 350212 China

**Keywords:** Tumor-associated macrophage, Prostate cancer, Machine learning, Cancer subtype, Single-cell RNA-seq

## Abstract

**Background:**

The tumor microenvironment (TME) encompasses a variety of cells that influence immune responses and tumor growth, with tumor-associated macrophages (TAM) being a crucial component of the TME. TAM can guide prostate cancer in different directions in response to various external stimuli.

**Methods:**

First, we downloaded prostate cancer single-cell sequencing data and second-generation sequencing data from multiple public databases. From these data, we identified characteristic genes associated with TAM clusters. We then employed machine learning techniques to select the most accurate TAM gene set and developed a TAM-related risk label for prostate cancer. We analyzed the tumor-relatedness of the TAM-related risk label and different risk groups within the population. Finally, we validated the accuracy of the prognostic label using single-cell sequencing data, qPCR, and WB assays, among other methods.

**Results:**

In this study, the TAM_2 cell cluster has been identified as promoting the progression of prostate cancer, possibly representing M2 macrophages. The 9 TAM feature genes selected through ten machine learning methods and demonstrated their effectiveness in predicting the progression of prostate cancer patients. Additionally, we have linked these TAM feature genes to clinical pathological characteristics, allowing us to construct a nomogram. This nomogram provides clinical practitioners with a quantitative tool for assessing the prognosis of prostate cancer patients.

**Conclusion:**

This study has analyzed the potential relationship between TAM and PCa and established a TAM-related prognostic model. It holds promise as a valuable tool for the management and treatment of PCa patients.

**Supplementary Information:**

The online version contains supplementary material available at 10.1007/s00262-024-03633-5.

## Introduction

Prostate cancer (PCa) is currently the most commonly diagnosed cancer among males (29%), and is the second leading cause of cancer-related deaths in the USA [1]. It is estimated that there will be 288,300 new cases and approximately 34,700 deaths in 2023 [2]. Although the majority of cases involve indolent or slow-progressing tumors, still about 20% of patients will develop high-aggressive and lethal forms of prostate cancer [3]. In recent years, despite our deeper understanding of the molecular pathogenesis of PCa, [4] there remains a quest to discover novel clinically useful multigene signature as molecular stratification biomarkers, which will provide predictive biomarkers for PCa and timely make the decision for therapy selection [5].

The tumor microenvironment (TME) contains diverse population of non-tumor stromal cell that play a role in tumor immune evasion and immunosuppression [6]. The human prostate tumor microenvironment consists of various stromal cells or extracellular matrix, including prostate epithelial cells, myoepithelial cells, fibroblasts, pericytes, endothelial cells, immune cells, adipocytes, neuronal cells, and neuroendocrine cells [7]. Among these, tumor-associated macrophages (TAMs), a crucial component of the tumor microenvironment, typically impact tumor growth through activities such as promoting neovascularization, secreting cytokines that inhibit tumor cell apoptosis, stimulating tumor cell proliferation and invasion, and inducing immunosuppression [8]. Based on the concept of classical and alternative activation, macrophages undergo polarization into distinct categories known as M1 and M2 [9, 10]. Studies suggest that TAMs predominantly exhibit an M2-like phenotype, characterized by immunosuppressive states and promoting tumor progression [11]. Therefore, it can be served as a strategy to block the downstream signaling pathways of M2 macrophages or inhibit M2-secreted factors that facilitate tumor development and progression for PCa intervention.

Despite extensive studies focusing on prostate cancer (PCa) and tumor-associated macrophages (TAMs), the overall characterization of TAMs and its relationship with PCa prognosis and immunotherapy response remain poorly understood. Therefore, we obtained prostate cancer single-cell RNA sequencing (scRNA-seq) data and transcriptome data from accessible databases. In this study, we conducted subclusters analysis of PCa and identified TAMs-based risk signature for PCa. We further investigated the clinical relevance of the TAM-based risk signature of PCa and analzyed the immune landscape and responsiveness to immunotherapy underlying the TAM-based signature. Ultimately, we integrated the TAM-based risk genes with clinical-pathological signature to establish a novel nomogram, aiming to provide a more precise clinical application for improving PCa patient prognosis and offering more individualized therapeutic strategies.

## Materials and methods

### Data sources

The single-cell sequencing data for this study were obtained from GSE193337, which includes 4 samples of prostate cancer tissue and 4 samples of adjacent benign tissue. Data quality control and dimensionality reduction criteria were set as follows: a minimum gene expression threshold of 250 in individual cells, inclusion of genes expressed in at least 3 cells, mitochondrial content below 15%, and a unique molecular identifier (UMI) count less than 50,000.

The training dataset for machine learning was derived from the TCGA-PRAD dataset, comprising gene expression data and clinical information from 494 prostate cancer samples. The test dataset was collected from GSE46602, GSE70768, GSE70769, GSE116918, and MSKCC-PRAD. These datasets were merged using the "sva" data package, resulting in a combined dataset containing gene expression data and clinical information for a total of 1027 prostate cancer patients. External validation data were sourced from SUPRAD, consisting of 136 cases of prostate cancer patients.

### TAM subpopulations in single-cell sequencing data

After quality control on the single-cell sequencing data, UMAP dimensionality reduction was performed on the entire dataset based on the top 2000 highly variable genes in each cell. This process ensured that there was no mixing between subpopulations. No batch correction was required. Subsequently, annotation of subpopulations was carried out with dim = 30 and resolution = 0.1. For the annotation of tumor-associated macrophages (TAMs), three marker genes, namely CD68, CD86, and CD163, were utilized. Further UMAP dimensionality reduction was performed on TAM clusters. The comparison of different clusters was conducted based on the criteria of logFC = 0.5, Minpct = 0.35, and *P* value < 0.05. This was done to identify marker genes for each TAM cluster (refer to Supplementary File 1). KEGG analysis was employed to investigate the cellular pathways involving the marker genes. CopyKAT R package was used to analyze the copy number variation (CNV) features of TAM subpopulations, distinguishing the proportions of tumor cells and normal cells within each subpopulation. Subsequently, the single-sample gene set enrichment analysis (ssGSEA) algorithm was employed to calculate the involvement of various tumor cell pathways in each TAM cluster. The clinical survival data from TCGA-PRAD, in conjunction with the TAM feature genes, were analyzed to investigate the relationship between each TAM cluster and prostate cancer (PCa) survival rates.

### Machine learning

In order to enhance the sensitivity and stability of TAM feature genes, we employed 10 machine learning algorithms to create 101 algorithm combinations. These comprehensive algorithms include Random Survival Forest (RSF), Elastic Net (Enet), Lasso, Ridge, Stepwise Cox, CoxBoost, plsRcox, SuperPC, Generalized Boosted Regression Model (GBM), and Survival Support Vector Machine (survival-SVM). The signature generation process is as follows: (a) Univariate Cox regression identified feature genes in the TCGA-PRAD cohort that could distinguish prostate cancer progression status (progression includes death or tumor metastasis, biochemical recurrence). (b) Subsequently, 101 algorithm combinations were applied to fit predictive models on the feature genes in the TCGA-PRAD cohort using a leave-one-out cross-validation (LOOCV) framework. (c) The predictive models were then applied to calculate predictions on five validation datasets (GSE46602, GSE70768, GSE70769, GSE116918, MSKCC-PRAD). (d) For each model, the Harrell's Concordance Index (C-index) was calculated across all validation datasets, and the model with the highest average C-index was considered the best (Supplementary 2).

### Prognostic model

For the selected feature genes identified through machine learning, and in conjunction with clinical survival data from various datasets, the six datasets were randomly divided into training and internal validation groups. In the training group, based on 9 TAM genes, a multivariate Cox analysis was conducted using the survival package to construct a risk model (Supplementary 3). The formula used for calculation is as follows: riskScore = βi*MGi, where βi represents the expression level of risk genes, and MGi represents the gene expression coefficients selected by machine learning. Using the median risk score as a cutoff, patients in the training group were stratified into high- and low-risk groups, biochemical relapse free was considered as a survival endpoint, the survival outcomes of these two groups were compared to validate the accuracy of the model, and Kaplan–Meier (KM) curves were plotted. The accuracy of the risk model was further validated in the internal validation group and an external dataset, SUPRAD (Supplementary 4 and 5). Combining the prognostic model with clinical features, column plots were generated using the "RMS" package. Calibration curves and time-dependent ROC curves were employed to assess the accuracy of the risk scoring model and the column plots.

### TAM feature gene's immune function

To further explore the tumor immune microenvironment, the "maftools" package in R was used to process TCGA mutation gene data. Gene copy number variations in prostate cancer were analyzed using Perl. Mutation analysis of TAM feature genes in TCGA-PRAD was conducted, and mutual exclusivity and co-linearity analyses were performed on the TOP 10 mutated genes and TAM feature genes. The "CIBERSORT" package was utilized to calculate immune cell infiltration in TCGA-PRAD and analyze the correlation between TAM feature genes and immune cells.

### qPCR and western blot

RNA extraction was carried out using the TRIzol reagent kit (Invitrogen, Carlsbad, CA) from various cells such as RWPE-1, 22RV1, and DU145. After determining RNA concentration using a spectrophotometer, the extracted RNA was converted to cDNA using a reverse transcription kit (TransGen Biotech). Specific primers for target genes were designed in NCBI (refer to Supplementary File 6) and synthesized by Shangya Biotechnology Co., Ltd. (Fuzhou, China). qPCR amplification was performed by mixing an appropriate amount of cDNA, gene-specific primers, ddH2O, and 2 × Taq Pro Universal SYBR qPCR Master Mix. GAPDH was used as an internal reference, and the relative expression level of target genes was calculated using the 2^−ΔΔCt^ method. For protein extraction, cells like RWPE-1, 22RV1, DU145, etc., were treated with protease inhibitors, and protein was extracted using RIPA buffer. Protein concentration was determined using the BCA protein concentration assay kit, and protein levels were analyzed following standard Western blot (WB) procedures.

### APOE function

In the GWAS database (https://gwas.mrcieu.ac.uk/), "prot-a-132" represents APOE which was used as the exposure factor for mendelian randomization (MR), “prot-a-1937” and “prot-a-680” represents M2 macrophage cell were used as the outcome. Correlation analysis was conducted to select SNPs strongly correlated with the exposure factor as instrumental variables, with a filtering criterion of *p* value < 5e − 06. Analyses included inverse variance weighted, weighted median, MR Egger, weighted mode, and simple mode to assess the association between APOE and M2 macrophage cell development (Supplementary File 7).

DU145 cells were cultured in six-well plates. When cell confluence reached above 70%, siAPOE reagent was added to the culture dish in conjunction with Lipofectamine 8000. siAPOE transfection plasmids were purchased from GimaGene (Suzhou, China) (refer to Supplementary Files 8, 9, 10). RNA was extracted 36 h after transfection, and protein was extracted 72 h after transfection. Both qPCR and WB were conducted to verify transfection efficiency. Scratch assay: After DU145 reached 90% confluence post-transfection, a uniform scratch was made using a bent inoculation needle. The cells were gently washed three times with PBS to remove detached cells, and serum-free medium was added. Cells were incubated at 37 °C in a 5% CO2 incubator. Cell migration was observed under an inverted microscope at 0 h and 24 h, and the closure rate was calculated for each group.

Cell migration assay: siAPOE-DU145 cell concentration was adjusted to 4 × 104 cells/300 μl. In the Transwell culture dish, 500 μl of medium containing 10% FBS was added to the lower chamber, and 300 μl of cell suspension was added to the upper chamber. Cells were incubated at 37 °C in a 5% CO_2_ incubator for 48 h. The upper chamber was removed, and the residual solution inside the chamber was discarded. The chamber was fixed in 1 ml of 4% paraformaldehyde for 20 min. After fixation, the chamber was placed upside down on filter paper and air-dried. Crystal violet staining was performed in a dark room for 30 min. The chamber was rinsed with PBS to remove excess dye, and cells were counted in at least 5 different fields under a microscope.

**Fig. 1:**
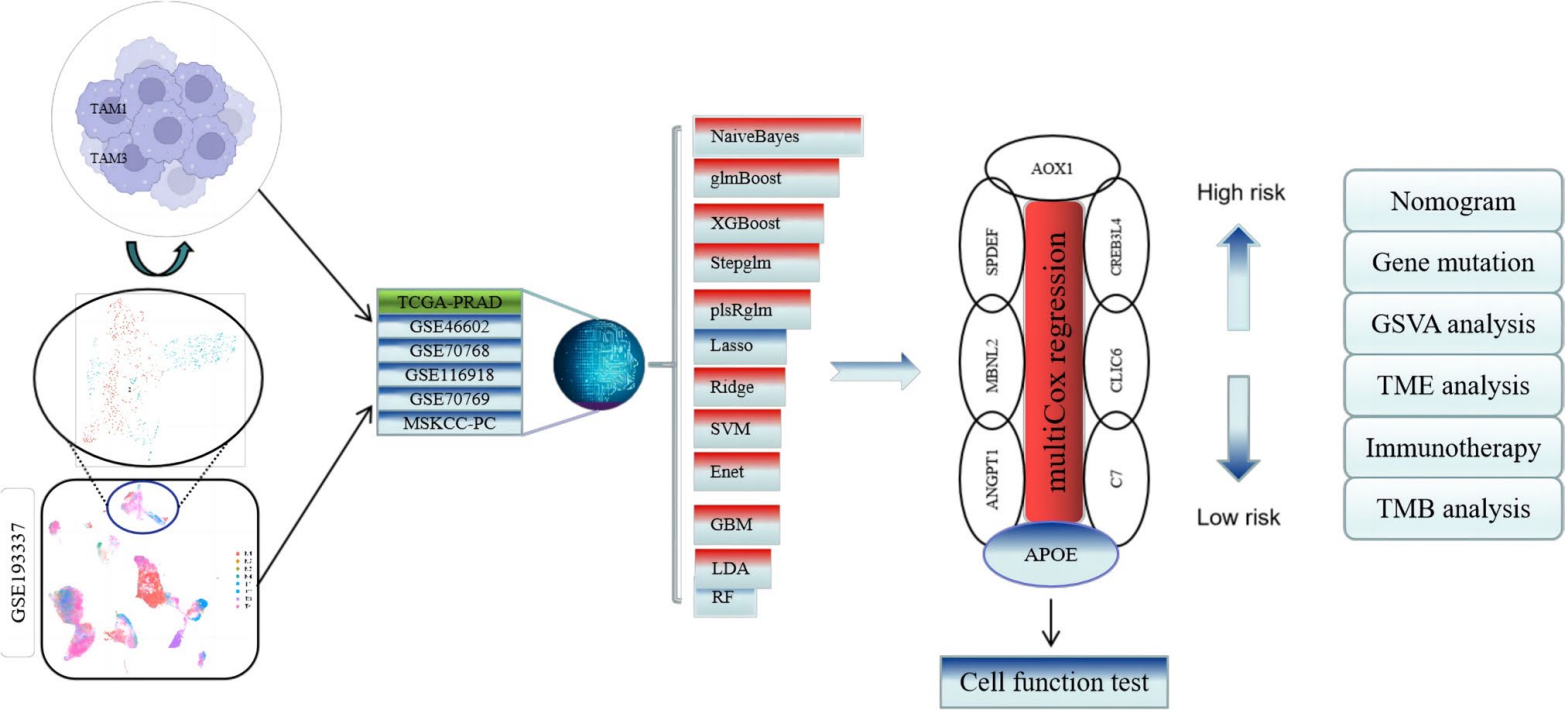
Study flowchart

This study used R-4.1.2 and Perl-5.32.1.1 for analysis. The R code was in supplementary file. *P* < 0.05 was considered statistically significant (*P value < 0.05; **p < 0.01; ***p < 0.001). The research workflow is shown in Fig. 1.

## Results

### Single-cell sequencing analysis

**Fig. 2:**
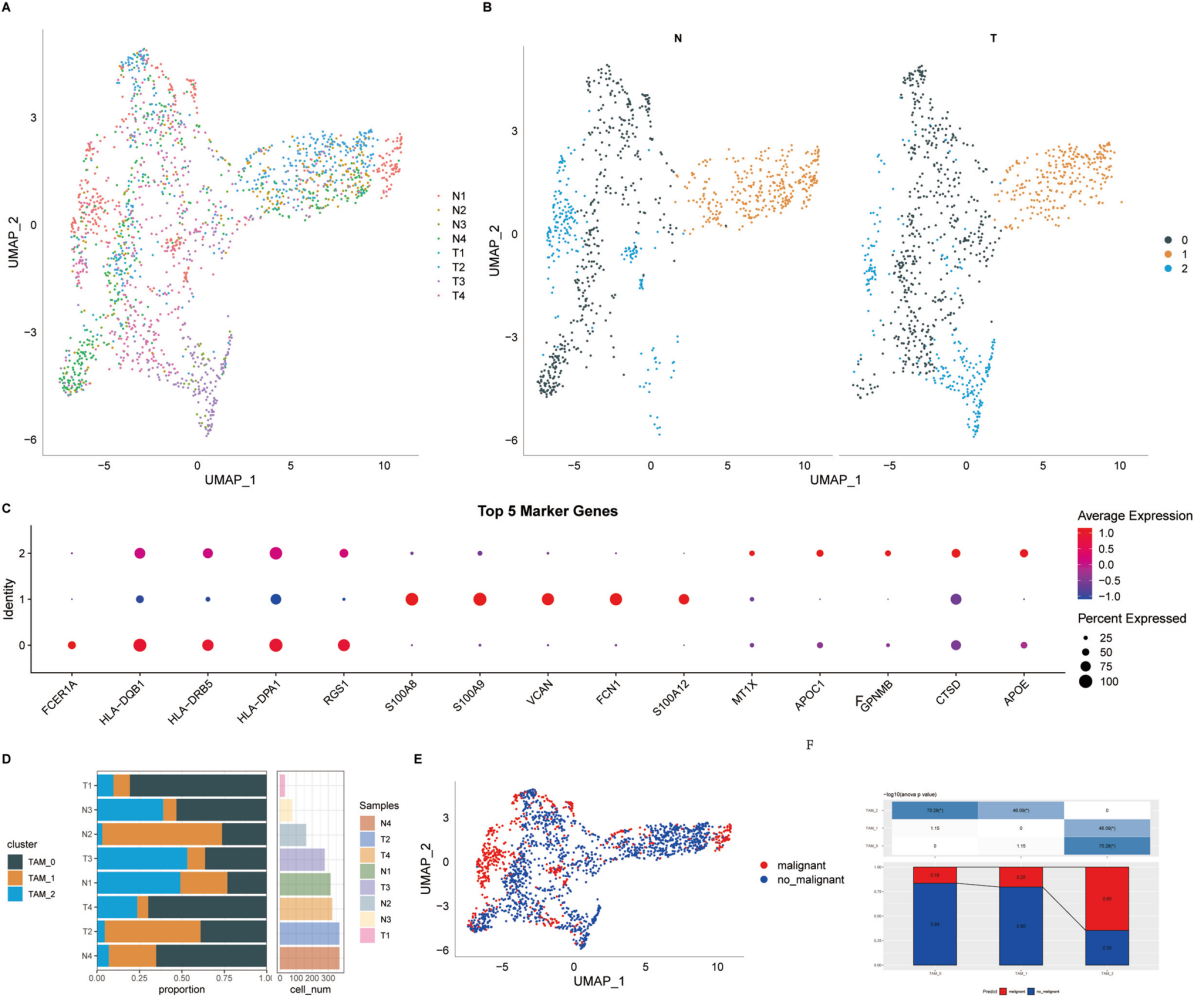
Identification of TAM Clusters in GSE19333. (**A**) TSNE plot showing the distribution of 8 samples. (**B**) Distribution of 3 TAM clusters in tumor cells and adjacent cells. (**C**) Top 5 marker genes for each TAM cluster. (**D**) Proportion and number of each TAM cluster in tumor and adjacent tissues. (**E**) TSNE plot showing the distribution of malignant and benign cells within TAM clusters. (**F**) Comparison of the proportions of TAM clusters in tumor and adjacent tissues. (**P < 0.05; ***P < 0.01; ****P < 0.001)

After performing quality control on the scRNA-seq data from 8 prostate-related samples, a total of 64,567 cells were obtained. Following normalization and dimensionality reduction based on gene expression values for individual cells, 18 cell subtypes were identified (Supplementary Fig. 1A). Using marker genes CD68, CD86, and CD163, 2 TAM subpopulations were identified, labeled as cluster 5 and cluster 13 (see Supplementary Fig. 1B). Further clustering and dimensionality reduction of cells from these 2 TAM subpopulations led to the identification of 3 TAM clusters (Supplementary Fig. 1C, D). Figure 2A shows the t-SNE plot of the distribution of the 8 single-cell sequencing samples, while Fig. 2B illustrates the distribution of the 3 TAM clusters within the samples. Among these 3 TAM clusters, 555 differentially expressed genes (DEGs) were identified. The top 5 DEGs from each cluster were considered as marker genes for these TAM clusters (Fig. 2C). TAM clusters are not entirely composed of prostate cancer malignant cells, the 3 TAM clusters consisted of 560 tumor cells and 1,367 normal cells (Fig. 2D, E). TAM_2 cluster had a higher proportion of malignant cells at 65%, while TAM_0 and TAM_1 had a higher proportion of benign cells (Fig. 2F).

### TAM cluster identification

**Fig. 3:**
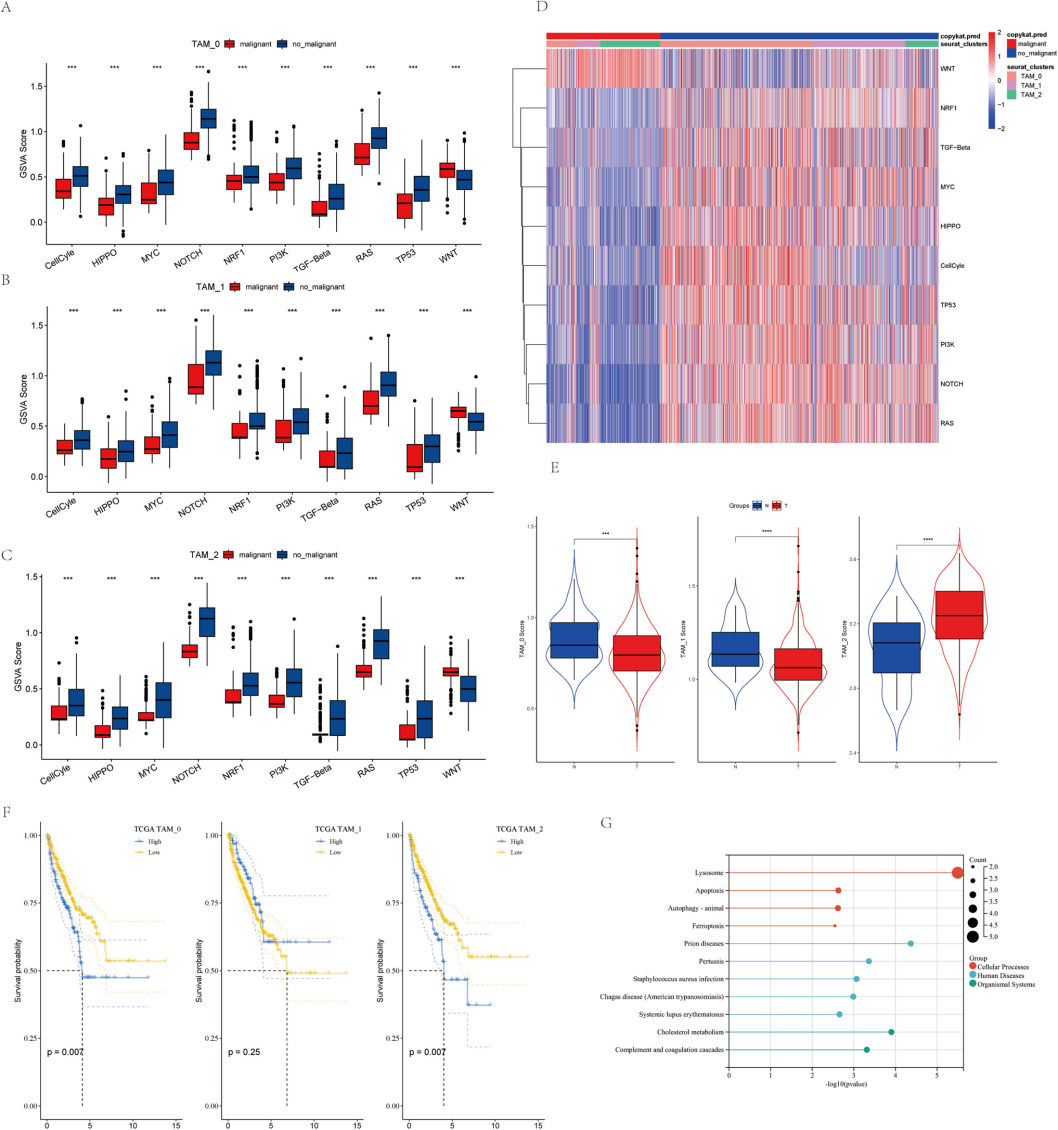
Features of Tumor-Related Pathways in TAM Clusters. (**A**) Comparison of GSVA scores for tumor-related pathways in TAM_0. (**B**) Comparison of GSVA scores for tumor-related pathways in TAM_1. (**C**) Comparison of GSVA scores for tumor-related pathways in TAM_2. (**D**) Heatmap showing enrichment scores of 10 tumor-related pathways in TAM cells. (**E**) Evaluation of pathway scores in TAM clusters in tumor and adjacent tissues. (**F**) Kaplan–Meier curves for high and low TAM scores in TAM_0 and TAM_2 clusters. (**G**) Biological pathways of feature genes in TAM_0 and TAM_2 clusters. (**P < 0.05; ***P < 0.01; ****P < 0.001)

To understand the relationship between TAM clusters and prostate cancer progression, we examined 10 cancer-related pathways within the 3 TAM clusters. The GSVA scores for these 10 pathways in different TAM clusters are displayed in Fig. 3A, B, C. Except for the WNT pathway, the other pathways were predominantly expressed in benign cells (Fig. 3D). However, there was no significant difference in GSVA scores between malignant and non-malignant cells in these 3 TAM clusters. To determine the relationship between each TAM cluster and patient prognosis, we first calculated ssGSEA scores for marker genes of each TAM cluster based on the TCGA-PRAD cohort. The scores were lower in tumor samples than in normal samples for TAM_0 and TAM_1, but the opposite trend was observed for TAM_2 (Fig. 3E). Using the median ssGSEA score as a cutoff, we divided the TCGA-PRAD samples of each TAM cluster into high and low TAM groups. The low TAM score group in TAM_0 and TAM_2 was significantly better disease-free survival than the high TAM group, whereas TAM_1 showed no significant correlation with prognosis in TCGA-PRAD (Fig. 3F). These findings suggest that the clustering of TAM_0 and TAM_2, based on 161 feature genes, might be associated with the development of prostate cancer. These genes primarily participate in biological processes, such as autophagy, ferroptosis, and lysosomal transport (Fig. 3G).

### Machine learning for TAM feature genes identification

To identify a gene set most relevant to prostate cancer development from the 161 TAM feature genes, we used 10 machine learning algorithms to form 101 algorithm combinations. These were evaluated based on the TCGA-PRAD as the training group and GSE116918, GSE46602, GSE70768, GSE70769, and MSKCC datasets as the test group. After comparing the different combinations, we selected "Lasso + RF" as the method for screening TAM feature genes. This approach yielded 9 genes that could effectively predict tumor progression (metastasis or progression to CRPC stage).

**Fig. 4:**
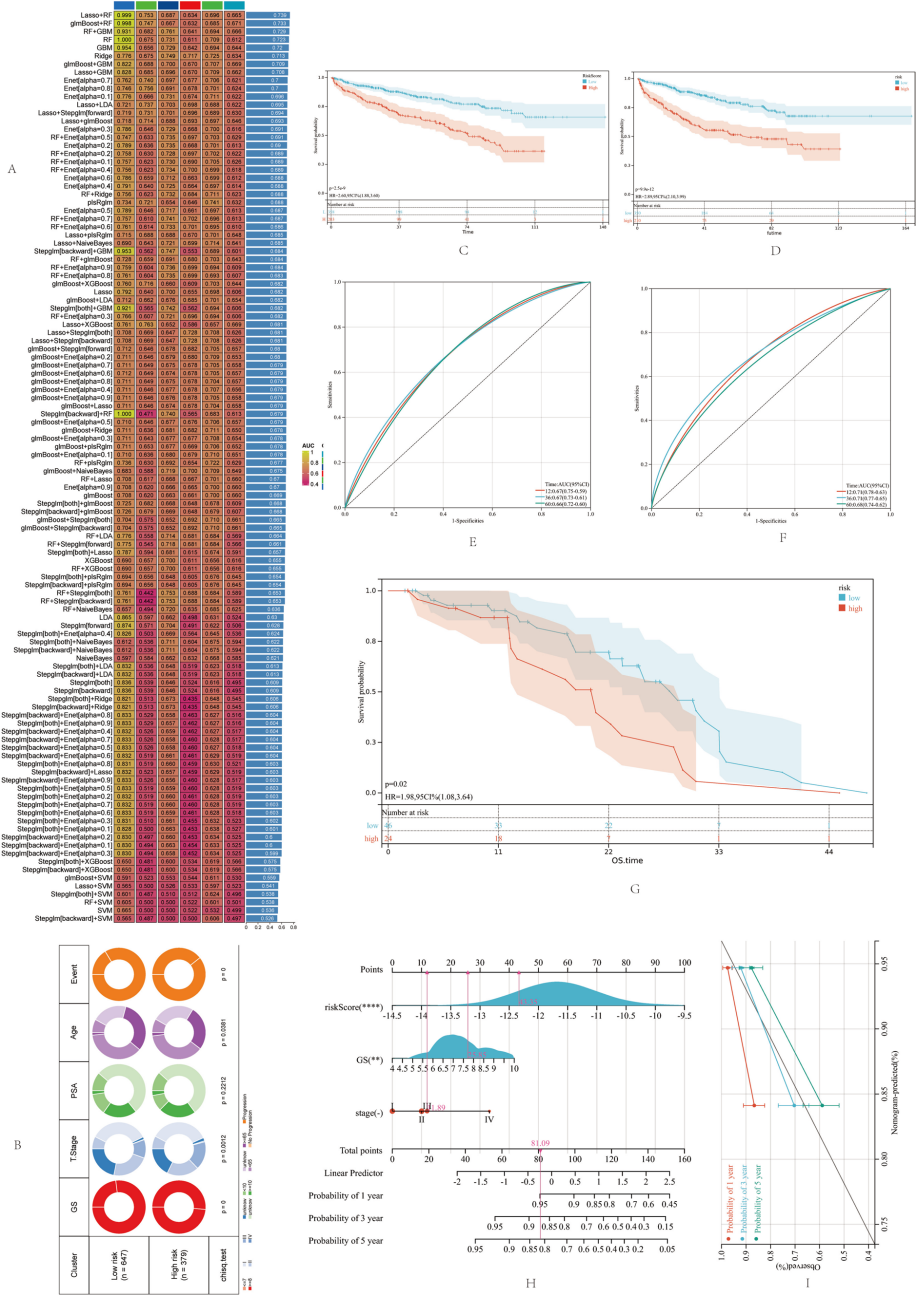
Machine Learning Selection of TAM Feature Genes. (**A**) AUC values for TAM feature genes selected by 10 machine learning methods for predicting prostate cancer status. (**B**) Clinical characteristics of patients in 6 prostate cancer datasets used for prognostic model construction. (**C**) Kaplan–Meier curves for high- and low-risk groups in the training dataset. (**D**) Kaplan–Meier curves for high- and low-risk groups in the internal validation dataset. (E) AUC values for the prognostic model in the training dataset at 1-, 3-, and 5-years. (**F**) AUC values for the prognostic model in the internal validation dataset at 1-, 3-, and 5-years. (**G**) Kaplan–Meier curves for high- and low-risk groups in the external validation dataset. (**H**) Nomogram model. (**I**) Calibration curves at 1-, 3-, and 5-years

Subsequently, we merged TCGA-PRAD, GSE116918, GSE46602, GSE70768, GSE70769, and MSKCC datasets, randomly dividing them into training and internal test groups (note that these are different from the machine learning groups). Using the patients' disease progression time, we built a prognosis model in the training group based on multiple Cox regression analysis. The risk score calculation formula for the prognosis model was as follows: RiskScore = ANGP1*(− 0.282) + AOX1*(− 0.404) + APOE*0.142 + C7*(− 0.255) + CLIC6*(− 0.673) + CREB3L4*0.092 + MBNL2*(− 0.52) + SPDEF*(− 0.443) + ST6GALNAC4*0.22, with a best cutoff value of − 11.50. This allowed us to divide the patients in the training group into high- and low-risk groups. The clinical characteristics of the two groups are shown in Fig. 4B. We observed a significant difference in disease progression between the two groups in the training group (*P* < 0.001), with AUC values of 0.67, 0.67, and 0.66 for the 1, 3, and 5-year survival models, respectively (Fig. 4C, E). Similarly, in the internal test group, patients in the high-risk group had significantly worse prognosis, with AUC values of 0.71, 0.71, and 0.68 for the 1, 3, and 5-year survival models, respectively (Fig. 4D, F). Most importantly, in the external test group, patients in the low-risk group, as calculated by the risk score, also exhibited significantly better prognosis (Fig. 4G). By combining the gene signature and clinical stage, we created a nomogram for the model. In predicting patient survival, the feature score formed by the 9 TAM-related genes was independent of other clinical pathological features, with a C-index of 0.71 (Fig. 4H). In the calibration curve, the X-axis represented the predicted outcomes of PCa progression using nomograms, while the Y-axis represented the actual PCa progression. The nomograms showed similar predictive abilities to actual disease progression (Fig. 4I).

### Immune function of TAM feature genes

**Fig. 5:**
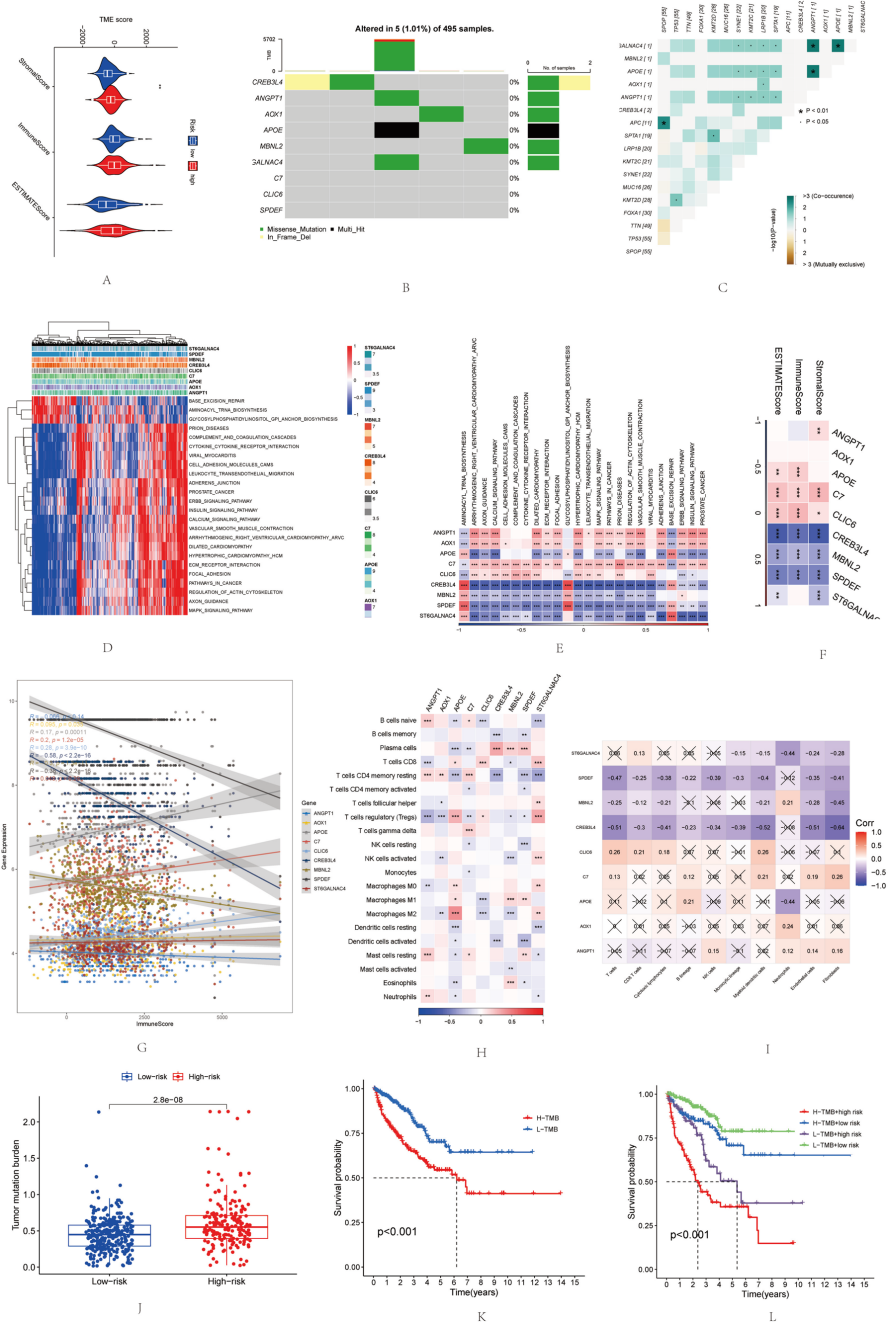
Immune Correlation of TAM Feature Genes. (**A**) Differences in ssGSEA scores between high- and low-risk groups. (**B**) Mutation status of TAM feature genes in TCGA-PRAD. (**C**) Co-expression of TAM feature genes with the top 10 mutated genes in TCGA-PRAD. (**D**) Heatmap showing the correlation between TAM feature genes and pathways. (**E**) Heatmap of enrichment scores for key pathways. (**F**, **G**) Association between 9 TAM feature genes and immune scores. (**H**, **I**) Association between 9 TAM feature genes and immune cell activity levels. (**J**) Differences in TMB between high- and low-risk groups. (**K**) Kaplan–Meier curves for high and low TMB groups. (**L**) Kaplan–Meier curves combining TMB levels and risk scores in TCGA-PRAD. (*P < 0.05; **P < 0.01; ****P < 0.001)

Estimate and CIBERSORT were used to evaluate the proportion of immune cells in patient samples. Patients in the high-risk group had higher stromal cell infiltration scores (Fig. 5A). Stromal components in the tumor microenvironment (TME) may better indicate patient prognosis. Subsequently, we examined the copy number variation (CNV) mutations of the 9 risk signature genes. Results indicated that these 9 TAM genes did not exhibit high-frequency CNV mutations in TCGA-PRAD (Fig. 5B). We analyzed the co-occurrence probability of these 9 TAM genes with the 10 most highly mutated genes in TCGA-PRAD. As shown in Fig. 5C, there was no significant probability of mutation co-occurrence for these 9 genes, except for ANGP1, APOE, which showed significant co-occurrence with genes like SYNE1, KMT2C, LRP1B.

Additionally, we analyzed the potential pathways associated with each TAM feature gene, as depicted in Fig. 5D, E. This included 24 biological pathways, such as the MAPK SIGNALING PATHWAY, LEUKOCYTE TRANSENDOTHELIAL MIGRATION, and ECM RECEPTOR INTERACTION. ANGPT1, APOE, C7, and CLIC6 showed significant positive correlations with stromal scores, immune scores, and ESTIMATE scores. In contrast, CREB3L4, MBNL2, SPDEF, and ST6GALNAC displayed significant negative correlations with these scores (Fig. 5F, G).

An analysis of the relationship between TAM feature genes and immune cells revealed that SPDEF, MBNL2, and CREB3L4 were negatively correlated with most immune cells, while CLIC6 was significantly positively correlated with most immune cells. APOE showed the most significant positive correlation with M2 macrophages (Fig. 5H, I). Tumor mutational burden (TMB), an indicator of high gene mutation frequency in tumor cells, was assessed. Patients in the high-risk group had significantly higher TMB than those in the low-risk group (Fig. 5J). Lower TMB corresponded to more favorable survival outcomes (Fig. 5K). Combining risk scores and TMB revealed that patients in the "high-risk group—high TMB group" had the worst prognosis, while those in the "low-risk group—low TMB group" had the most favorable prognosis (Fig. 5L).

### Function of APOE

**Fig. 6:**
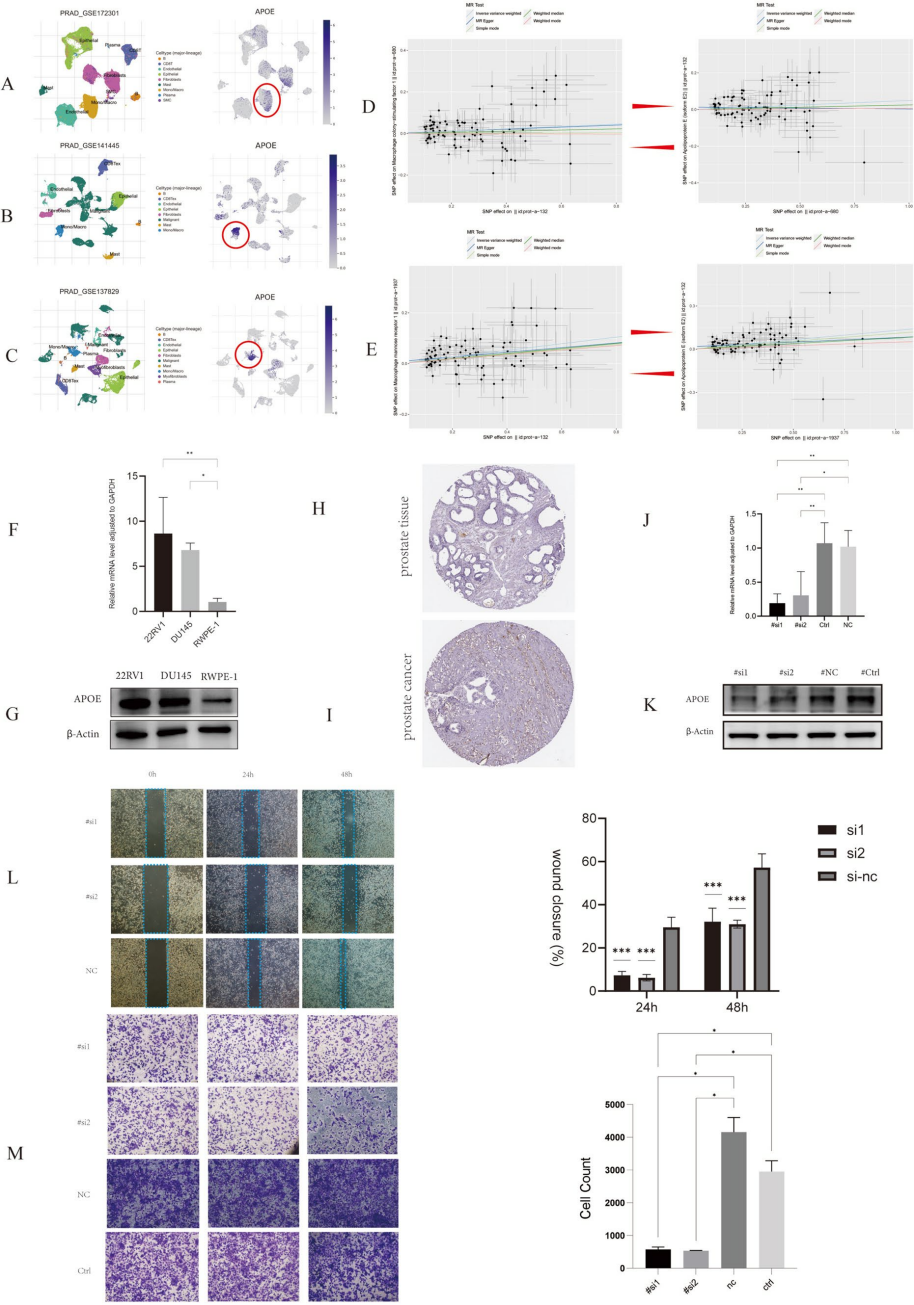
Role of APOE in Prostate Cancer. (**A**, **B**, **C**) Distribution of APOE in the prostate cancer TME. (**D**, **E**) Scatter plots of genetic associations with APOE against the macrophage factor level. The slopes of each line represent the causal association for each method. The blue line represents the inverse‐variance weighted estimate, the green line represents the weighted median estimate, and the dark blue line represents the Mendelian randomization‐Egger estimate. (**F**, **G**) mRNA and protein expression levels of APOE in different prostate cancer cell lines. (**H**, **I**) Immunohistochemistry results of APOE in cancer and adjacent tissues. (**J**, **K**) mRNA and protein expression levels of siAPOE-DU145. (**L**, **M**) Scratch and migration assays of DU145 cells after si-1, si-2, si-nc transfection

Through single-cell sequencing analysis using immusc-vue (https://immucanscdb.vital-it.ch/) in GSE172301, GSE137829 and GSE14144, we found that APOE was predominantly expressed in PCa macrophages (Fig. 6A, B, C). Macrophage colony-stimulating factor 1 and Macrophage mannose receptor 1 are important drivers of M2 macrophages. Before MR analysis, we first confirmed that there was no significant heterogeneity or pleiotropy among instrumental variables (Supplementary Files 6). Forward MR Analysis found that with increasing APOE expression, Levels of Macrophage colony-stimulating factor 1 and Macrophage mannose receptor 1 were also increased. Meanwhile, Macrophage colony-stimulating factor 1 and Macrophage mannose receptor 1 were also found to increase APOE levels by reverse MR analysis, reconfirming the close link between APOE and M2 macrophages (Fig. 6D, E). Based on previous research and preliminary analysis, we focus on the function of APOE gene in prostate cancer. Using qRT-PCR and Western blot validation, we found that compared to normal prostate epithelial cells (RWPE-1), APOE expression levels were higher in DU145 cells and 22RV1 cells (Fig. 6F, G). Data from the HPA database also showed positive APOE expression in prostate cancer, with no protein expression in normal prostate tissue (Fig. 6H, I). Following successful knockdown of APOE using siAPOE in DU145 cells (Fig. 6J, K), we assessed cell migration ability through Transwell migration and scratch assays. The results showed that DU145 cells in si-1 and si-2 transfection groups exhibited significantly reduced migration ability compared to the control group. The Transwell chamber migration assay confirmed the positive effect of APOE on cell migration (Fig. 6L, M).

## Discussion

TAM is the crucial component of immune infiltration in prostate cancer. TAMs can differentiate into two functional cell types under the influence of microenvironmental signals: the pro-inflammatory M1 phenotype and the anti-inflammatory M2 phenotype. These TAMs play a regulatory role in the process of tumor progression [12]. M2-type macrophages are known to infiltrate extensively in various cancer tissues, including gastric cancer, breast cancer, colon cancer, and bladder cancer. The degree of M2 macrophage infiltration is positively correlated with tumor staging, recurrence, and poor prognosis. These cells can promote cancer cell proliferation, invasion, enhance angiogenesis, and contribute to immune tolerance. In certain cases, TAMs can also accelerate resistance to standard therapies and immunotherapies. The depletion of TAMs has shown potential in improving the effectiveness of treatments, such as docetaxel in castration-resistant prostate cancer [13]. Therefore, developing clinical drugs that target the depletion of TAMs or their repolarization toward an anti-tumor state remains an urgent challenge. However, the complexity of the interactions between cancer cells and infiltrating macrophages has hindered progress in this area.

With the widespread application of single-cell RNA sequencing technology, more detailed insights into the functions and morphology of macrophages in prostate cancer have been discovered. Qian et al. [14] suggested that monocyte-derived macrophages play significant roles in promoting bone metastasis growth and drug resistance in prostate cancer. In contrast, tissue-resident macrophages do not directly promote bone metastasis but significantly enhance resistance to castration therapy for metastatic prostate cancer. In this study, single-cell RNA sequencing was employed to analyze the transcriptional landscape of TAMs in human prostate cancer. This approach identified a subpopulation of macrophages characterized by dysregulated transcriptional pathways related to lipid metabolism. Diletta Di Mitri et al. [15] found a lipid-related subset of TAMs that positively correlated with prostate cancer progression. Notably, these TAMs were found to promote cancer cell migration through the release of lipid-loaded CCL6.

Furthermore, this research conducted a systematic characterization and classification of TAMs in prostate cancer based on scRNA-seq data. Three distinct TAM clusters were identified based on the expression of CD68, CD86, and CD163 markers [16]. Among these clusters, TAM_0 and TAM_2 were found to be significantly associated with prostate cancer prognosis based on clinical features from TCGA-PRAD. It was observed that the GSVA scores of prostate cancer patients within the TAM_2 cluster were significantly higher than those of normal individuals, suggesting that TAM_2 may represent a subtype of macrophages associated with promoting tumor progression, resembling the traditional M2 macrophage function. Pathway analysis of the TAM_2 cluster revealed a concentration of biological pathways related to iron death, apoptosis, and lysosome formation. However, the clinical utility and feasibility of a large number of TAM feature genes were limited. Therefore, various machine learning methods were employed to screen TAM feature genes.

Researchers such as Yu-Ling Sun [17] and Haitao Niu [18] have previously combined multiple machine learning approaches to identify feature genes in different types of cancers, resulting in more precise prognostic models. Considering the prognostic value of the two TAM clusters, this study also used machine learning to select 9 TAM feature genes in TCGA-PRAD, which were effective in distinguishing the progression status of prostate cancer in five other datasets. A significant difference in this study is that, unlike previous research [19], TCGA-PRAD was not exclusively used as the training group in subsequent modeling. Instead, the training and test datasets from six different sources were merged and randomly divided into training and internal test groups. Based on the training group, a risk signature with 9 genes related to TAMs was developed. This approach, which minimized model robustness, was validated both internally and externally, demonstrating the accuracy of the TAM risk signature. The prognostic model consisted of 6 protective genes (ANGP1, AOX1, C7, CLIC6, MBNL2, and SPDEF) and 3 risk genes (APOE, CREB3L4, and ST6GALNAC4). In this study, no CNV mutations were observed in the 9 TAM feature genes. However, APOE exhibited co-occurring mutations with SYNE1, KMT2C, and LRP1B. These gene mutations could potentially impact the activity or function of key proteins in prostate cancer, affecting its development and progression. Additionally, APOE was found to have the closest association with M2 macrophage levels among the TAM feature genes, warranting further investigation.

Apolipoprotein E (ApoE) is a polymorphic molecule involved in lipid transport and lipid metabolism, mediating the distribution/re-distribution of lipids in tissues and cells. It has been linked to degenerative diseases, such as atherosclerosis (AS), coronary heart disease (CHD), and Alzheimer's disease (AD) [20]. In recent years, ApoE has been identified as a potential diagnostic or prognostic marker in many cancers. There is a significant relationship between ApoE and the tumor microenvironment (TME) [21]. ApoE can mediate inflammation and immune responses in the TME, stimulate angiogenesis, and participate in tumor progression, proliferation, and metastasis. For example, Zheng et al. [22] found that ApoE in extracellular vesicles derived from M2 macrophages in gastric cancer activates the PI3K-Akt signaling pathway, promoting gastric cancer cell migration and playing a crucial regulatory role in the tumor microenvironment. Bancaro et al. [23] discovered increased expression of APOE in prostate cancer and its binding to TREM2 on immunosuppressive neutrophils, leading to their senescence and death, revealing another mechanism of tumor immune evasion. Wong et al. [24] identified an increase in C1QB + TREM2 + APOE + -M2 macrophages in the prostate cancer TME, which may be closely associated with the invasive phenotype of prostate cancer. Masetti et al. [25] found a positive correlation between the abundance of lipid-rich tumor-associated macrophages (TAMs) and tumor invasiveness in male prostate cancer. Ioannidou et al. [26] observed that genetically predicted lipoprotein(a) concentrations were associated with an increased risk of prostate cancer. Based on these findings, it is hypothesized that M2 macrophages in prostate cancer release APOE, contributing to the regulation of the tumor microenvironment.

However, there are limitations to this study. Firstly, retrospective data from public databases were used to generate TAM clusters and TAM-based risk signatures. Therefore, it is essential to validate these findings in future prospective and multicenter prostate cancer cohorts. Secondly, the study focused on the potential prognostic value of TAM-based risk signatures and requires further laboratory research to explore the specific role of this signature in prostate cancer development.

## Conclusion

This study systematically characterized three TAM populations in prostate cancer, demonstrating their diversity. DEGs in these three clusters were found to be enriched in signaling pathways related to prostate cancer autophagy, iron death, apoptosis, and others. Two of these clusters were significantly associated with prostate cancer prognosis. Through the application of machine learning, nine TAM feature genes were identified, and a prognostic risk signature was constructed. Additionally, a nomogram was developed by associating TAM feature genes with clinical pathological features, providing a quantitative method for clinicians to assess the prognosis of prostate cancer patients.

### Supplementary Information

Below is the link to the electronic supplementary material.

Supplementary file1 (PDF 147 kb)

Supplementary file2 (PDF 4706 kb)

Supplementary file3 (PDF 2127 kb)

Supplementary file4 (XLSX 246 kb)

## Data Availability

The datasets presented in this study can be found in online repositories. The names of the repository/repositories and accession number(s) can be found in the article/Supplementary Material.

## References

[1] Siegel RL, Miller KD, Wagle NS (2023). Cancer statistics, 2023. CA Cancer J Clin.

[2] Saha A, Kolonin MG, Digiovanni J (2023). Obesity and prostate cancer - microenvironmental roles of adipose tissue. Nat Rev Urol.

[3] Chang AJ, Autio KA, Roach M (2014). High-risk prostate cancer-classification and therapy. Nat Rev Clin Oncol.

[4] Adamaki M, Zoumpourlis V (2021). Prostate cancer biomarkers: from diagnosis to prognosis and precision-guided therapeutics. Pharmacol Ther.

[5] Attard G, Parker C, Eeles RA (2016). Prostate cancer. Lancet.

[6] Xiang X, Wang J, Lu D (2021). Targeting tumor-associated macrophages to synergize tumor immunotherapy. Signal Transduct Target Ther.

[7] Lecoultre M, Dutoit V, Walker PR (2020). Phagocytic function of tumor-associated macrophages as a key determinant of tumor progression control: a review. J Immunother Cancer.

[8] Chen S, Saeed A, Liu Q (2023). Macrophages in immunoregulation and therapeutics. Signal Transduct Target Ther.

[9] Wang J, Long R, Han Y (2022). The role of exosomes in the tumour microenvironment on macrophage polarisation. Biochim Biophys Acta Rev Cancer.

[10] Shu Y, Cheng P (2020). Targeting tumor-associated macrophages for cancer immunotherapy. Biochim Biophys Acta Rev Cancer.

[11] Chen D, Zhang X, Li Z (2021). Metabolic regulatory crosstalk between tumor microenvironment and tumor-associated macrophages. Theranostics.

[12] Shapouri-Moghaddam A, Mohammadian S, Vazini H, Taghadosi M, Esmaeili SA, Mardani F, Seifi B, Mohammadi A, Afshari JT, Sahebkar A (2018). Macrophage plasticity, polarization, and function in health and disease. J Cell Physiol.

[13] Cao H, Wang D, Gao R, Feng Y, Chen L (2022). Qi Ling decreases paclitaxel resistance in the human prostate cancer by reversing tumor-associated macrophages function. Aging Albany NY.

[14] Li XF, Selli C, Zhou HL, Cao J, Wu S, Ma RY, Lu Y, Zhang CB, Xun B, Lam AD, Pang XC, Fernando A, Zhang Z, Unciti-Broceta A, Carragher NO, Ramachandran P, Henderson NC, Sun LL, Hu HY, Li GB, Sawyers C, Qian BZ (2023). Macrophages promote anti-androgen resistance in prostate cancer bone disease. J Exp Med.

[15] Masetti M, Carriero R, Portale F, Marelli G, Morina N, Pandini M, Iovino M, Partini B, Erreni M, Ponzetta A, Magrini E, Colombo P, Elefante G, Colombo FS, den Haan JMM, Peano C, Cibella J, Termanini A, Kunderfranco P, Brummelman J, Chung MWH, Lazzeri M, Hurle R, Casale P, Lugli E, DePinho RA, Mukhopadhyay S, Gordon S, Di Mitri D (2022). Lipid-loaded tumor-associated macrophages sustain tumor growth and invasiveness in prostate cancer. J Exp Med.

[16] Trombetta AC, Soldano S, Contini P, Tomatis V, Ruaro B, Paolino S, Brizzolara R, Montagna P, Sulli A, Pizzorni C, Smith V, Cutolo M (2018). A circulating cell population showing both M1 and M2 monocyte/macrophage surface markers characterizes systemic sclerosis patients with lung involvement. Respir Res.

[17] Wang L, Liu Z, Liang R, Wang W, Zhu R, Li J, Xing Z, Weng S, Han X, Sun YL (2022). Comprehensive machine-learning survival framework develops a consensus model in large-scale multicenter cohorts for pancreatic cancer. Elife.

[18] Chu G, Ji X, Wang Y, Niu H (2023). Integrated multiomics analysis and machine learning refine molecular subtypes and prognosis for muscle-invasive urothelial cancer. Mol Ther Nucleic Acids.

[19] Kang Z, Sun JB, Lin F, Huang XY, Huang Q, Chen DN, Zheng QS, Xue XY, Xu N, Wei Y (2023). Subtype and prognostic analysis of immunogenic cell death-related gene signature in prostate cancer. Front Oncol.

[20] Miao G, Zhuo D, Han X (2023). From degenerative disease to malignant tumors: insight to the function of ApoE. Biomed Pharmacother.

[21] Tavazoie MF, Pollack I, Tanqueco R (2018). LXR/ApoE activation restricts innate immune suppression in cancer. Cell.

[22] Zhao Z, Zou S, Guan X (2018). Apolipoprotein E overexpression is associated with tumor progression and poor survival in colorectal cancer. Front Genet.

[23] Bancaro N, Cali B, Troiani M (2023). Apolipoprotein E induces pathogenic senescent-like myeloid cells in prostate cancer. Cancer Cell.

[24] Wong HY, Sheng Q, Hesterberg AB (2022). Single cell analysis of cribriform prostate cancer reveals cell intrinsic and tumor microenvironmental pathways of aggressive disease. Nat Commun.

[25] Masetti M, Carriero R, Portale F (2022). Lipid-loaded tumor-associated macrophages sustain tumor growth and invasiveness in prostate cancer. J Exp Med.

[26] Ioannidou A, Watts EL, Perez-Cornago A, Platz EA, Mills IG, Key TJ, Travis RC (2022). PRACTICAL consortium, CRUK, BPC3, CAPS, PEGASUS; Tsilidis KK, Zuber V. The relationship between lipoprotein A and other lipids with prostate cancer risk: a multivariable Mendelian randomisation study. PLoS Med.

