# Supplementary material for: Identification macrophage signatures in prostate cancer by single-cell sequencing and machine learning
Source: Cancer Immunol Immunother. 2024 Feb 13;73(3):41. doi: 10.1007/s00262-024-03633-5 (PMC10864475; doi:10.1007/s00262-024-03633-5)
Supplement: Supplementary file 1 — Supplementary file1 (PDF 147 kb) [file 262_2024_3633_MOESM1_ESM.pdf]

```
setwd('')
dir.create('results')
options(stringsAsFactors = F,check.bounds = F)
library(Seurat)
library(dplyr)
library(ggplot2)
library(magrittr)
library(gtools)
library(stringr)
library(Matrix)
library(tidyverse)
library(patchwork)
library(data.table)
library(RColorBrewer)
library(ggpubr)
library(ggsci)
#
dir_name=list.dirs('GSE193337_RAW/',full.names = F,recursive = F)
dir_name
datalist=list()
for (i in 1:length(dir_name)){
  dir.10x = paste0("GSE193337_RAW/",dir_name[i])
  my.data <- Read10X(data.dir = dir.10x)
  colnames(my.data)=paste0(dir_name[i],colnames(my.data))
  datalist[[i]]=CreateSeuratObject(counts = my.data, project = dir_name[i], min.cells = 3,
min.features = 250)
  datalist[[i]]$Samples=dir_name[i]
  datalist[[i]]$type=substr(dir_name[i],1,1)
}
names(datalist)=dir_name

for (i in 1:length(datalist)){
  sce <- datalist[[i]]
  sce[["percent.mt"]] <- PercentageFeatureSet(sce, pattern = "^MT-")# 计算线粒体占比
  sce[["percent.Ribo"]] <- PercentageFeatureSet(sce, pattern = "^RP[SL]")# 计算 rRNA 占比
  datalist[[i]] <- sce
  rm(sce)
}
sce <- merge(datalist[[1]],y=datalist[2:length(datalist)])
raw_cell=sce@meta.data
raw_count <- table(raw_cell$Samples)
raw_count
sum(raw_count)#12554
```

```
pearplot_befor<-VlnPlot(sce,group.by ='Samples',
                        features = c("nFeature_RNA", "nCount_RNA",
"percent.mt","percent.Ribo"),
                        pt.size = 0,
                        ncol = 4)
pearplot_befor
ggsave('results/pearplot_befor.pdf',pearplot_befor,height = 5,width = 15)
ggsave('results/pearplot_befor.jpg',pearplot_befor,height = 5,width = 15,dpi = 300)

sample_color<-pal_nejm(alpha = 0.5)(8)[1:8]
sample_color
Feature_ber1<-FeatureScatter(sce,feature1 = 'nFeature_RNA',
                             feature2 = 'nCount_RNA',
                             group.by = 'Samples',
                             cols = sample_color)
Feature_ber2<-FeatureScatter(sce,feature1 = 'percent.mt',
                             feature2 = 'nCount_RNA',
                             group.by = 'Samples',
                             cols = sample_color)
Feature_ber3<-FeatureScatter(sce,feature1 = 'percent.mt',
                             feature2 = 'nFeature_RNA',
                             group.by = 'Samples',
                             cols = sample_color)
Feature_ber1=Feature_ber1+theme(legend.position = 'none')
Feature_ber2=Feature_ber2+theme(legend.position = 'none')

Feature_ber<-ggarrange(Feature_ber1,Feature_ber2,Feature_ber3,ncol = 3,nrow = 1,widths =
c(1,1,1.2))
ggsave('results/Feature_cor.pdf',Feature_ber,height = 5,width = 17)
ggsave('results/Feature_cor.jpg',Feature_ber,height = 5,width = 17,dpi = 300)

datalist <- lapply(X = datalist, FUN = function(x) {
  x<-subset(x,subset =
              nFeature_RNA < 5000 &
              percent.mt < 15)
})
sce <- merge(datalist[[1]],y=datalist[2:length(datalist)])
clean_cell=sce@meta.data

clean_count <- table(clean_cell$Samples)
clean_count
sum(clean_count)#6410
pearplot_after <- VlnPlot(sce,group.by ='Samples',
                          features = c("nFeature_RNA", "nCount_RNA",
```

```
"percent.mt","percent.Ribo"),
                                                pt.size = 0,
                                                ncol = 4)
pearplot_after
ggsave('results/pearplot_after.pdf',Feature_ber,height = 5,width = 15)
ggsave('results/pearplot_after.jpg',Feature_ber,height = 5,width = 15,dpi = 300)

save(datalist,file = 'datalist.RData')
sce <- merge(datalist[[1]],y=datalist[2:length(datalist)])
sce <- NormalizeData(sce, normalization.method = "LogNormalize", scale.factor = 10000)
sce <- FindVariableFeatures(sce,
                                                   selection.method = "vst",
                                                   nfeatures = 2000,#筛选前 2000 个高变，可修改的
                                                   mean.cutoff=c(0.0125,3),
                                                   dispersion.cutoff =c(1.5,Inf))
sce <- ScaleData(sce, features =    rownames(sce))
sce <- RunPCA(sce, features = VariableFeatures(sce))

elbowplot <- ElbowPlot(sce, ndims=50, reduction="pca")
elbowplot
ggsave('results/elbowplot.pdf',elbowplot,height = 5,width = 5)

Dims <- 30
sce <- RunUMAP(sce, dims=1:Dims, reduction="pca")
raw.umap<-DimPlot(sce,group.by='Samples',
                              reduction="umap",
                              label = "T",
                              pt.size = 0.2,
                              label.size = 0)+
   ggtitle('')
raw.umap
ggsave('results/raw.umap.pdf',raw.umap,height = 7,width = 7)
library(clustree)
sce <- FindNeighbors(sce, dims = 1:Dims)
sce <- FindClusters(
   object = sce,
   resolution = c(seq(.1,1,.1))
)
colnames(sce@meta.data)
clustree(sce@meta.data, prefix = "RNA_snn_res.")

pdf('results/clust.snn_res.pdf',he=15,wi=15)
clustree(sce@meta.data, prefix = "RNA_snn_res.")
dev.off()
```

```
Resolution <- 0.8
sce <- FindNeighbors(object = sce, dims = 1:Dims)
sce <- FindClusters(object = sce, resolution = Resolution)
DefaultAssay(sce) <- "RNA"
#fibroblast:9,11,21
VlnPlot(sce,features = c('CD68','CD86','CD163'),pt.size = 0,group.by = 'seurat_clusters',ncol = 2)
library(randomcoloR)
allcolour <- c(pal_npg(alpha = 0.8)(9),
                    pal_igv(alpha = 0.8)(9),
                    pal_jama(alpha = 0.8)(7),
                    pal_jco(alpha = 0.8)(9),
                    pal_nejm(alpha = 0.8)(8))
length(table(sce@active.ident))
#28
mycolor1 = allcolour[1:length(table(sce$seurat_clusters))]

figs2b<-FeaturePlot(sce,
                          features =    c('CD68','CD86','CD163'),
                          pt.size = 0.3,reduction = 'umap',ncol = 2)
figs2a<-DimPlot(sce,cols =mycolor1 ,group.by = 'seurat_clusters',
                     reduction="umap",
                     label = "T",
                     pt.size = 0.3,
                     label.size = 5) +
  theme(axis.line = element_line(size=0.1, colour = "black"),
        #axis.text = element_blank(),
        #axis.title = element_blank(),
        axis.ticks = element_blank()
  ) +ggtitle('')

figs2ab<-ggarrange(figs2a,figs2b,nrow = 1,ncol = 2,widths = c(1,1),labels = c('A','B'))
figs2ab
table(sce$seurat_clusters)
#
save(sce,file = 'sce1.RData')

load('sce1.RData')
Idents(sce)='seurat_clusters'
sce<-subset(sce,idents =c())
Resolution <- 0.1
DefaultAssay(sce) <- "RNA"

sce <- FindNeighbors(object = sce, dims = 1:Dims)
sce <- FindClusters(object = sce, resolution = Resolution)
```

```
DefaultAssay(sce) <- "RNA"

VlnPlot(sce,features = c('CD68','CD86','CD163'),pt.size = 0,group.by = 'seurat_clusters',ncol = 2)

sce <- RunUMAP(sce,
               dims=1:Dims,
               reduction="pca",
               perplexity=30,
               max_iter=1000)
figs2c<-DimPlot(sce,cols =mycolor1 ,group.by = 'seurat_clusters',
                reduction="umap",
                label = "T",
                pt.size = 0.2,
                label.size = 5) +
  theme(axis.line = element_line(size=0.1, colour = "black"),
        #axis.text = element_blank(),
        #axis.title = element_blank(),
        axis.ticks = element_blank()
  ) +ggtitle('')

figs2d<-FeaturePlot(sce,
                    features =   c('CD68','CD86','CD163'),
                    pt.size = 0.1,reduction = 'umap',ncol = 2)
fig2cd<-ggarrange(figs2c,figs2d,nrow = 1,ncol = 2,widths = c(1,1),labels = c('C','D'))
fig2cd

figs2<-ggarrange(figs2ab,fig2cd,nrow = 2,ncol = 1)
ggsave('results/FigS2.pdf',figs2,height =15,width = 15)

Logfc = 0.5
Minpct = 0.35
DefaultAssay(sce) <- "RNA"
Idents(sce)<-'seurat_clusters'
sce.markers <- FindAllMarkers(object = sce,logfc.threshold = Logfc, min.pct = Minpct,only.pos = T)
sce.markers["pct.diff"]=sce.markers$pct.1-sce.markers$pct.2
sce.markers <- sce.markers[sce.markers$p_val_adj<0.05,]
length(unique(sce.markers$gene))
head(sce.markers)
write.table(sce.markers,'results/scRNA_marker_gene.txt',quote = F,row.names = F,sep='\t')

Top5 <- sce.markers %>% group_by(cluster) %>% slice_max(n =5, order_by = avg_log2FC)

Top5 <- intersect(unique(Top5$gene),rownames(sce@assays$RNA@meta.features))
```

```
sc_marker_dotplot <- DotPlot(object = sce, features = Top5,cols=c("blue", "red"),scale = T)+
  RotatedAxis()+ ggtitle("Top 5 Marker Genes")+
  theme(plot.title = element_text(hjust = 0.5)) +xlab('')

sc_marker_dotplot
ggsave('results/sc_marker_dotplot.pdf',sc_marker_dotplot,height = 7,width = 9)
bubble.df=as.matrix(sce[["RNA"]]@data[Top5,])
bubble.df=t(bubble.df)
bubble.df=as.data.frame(scale(bubble.df))
bubble.df$CB=rownames(bubble.df)
bubble.df=merge(bubble.df,
                data.frame(CB=rownames(sce@meta.data),
                           celltype=sce@meta.data$seurat_clusters),
                by = "CB")
bubble.df$CB=NULL

celltype_v=c()
gene_v=c()
mean_v=c()
ratio_v=c()
for (i in unique(bubble.df$celltype)) {
  bubble.df_small=bubble.df%>%filter(celltype==i)
  for (j in Top5) {
    exp_mean=mean(bubble.df_small[,j])
    exp_ratio=sum(bubble.df_small[,j] > min(bubble.df_small[,j])) / length(bubble.df_small[,j])
    celltype_v=append(celltype_v,i)
    gene_v=append(gene_v,j)
    mean_v=append(mean_v,exp_mean)
    ratio_v=append(ratio_v,exp_ratio)
  }
}
plotdf=data.frame(
  celltype=celltype_v,
  gene=gene_v,
  exp=mean_v,
  ratio=ratio_v)
plotdf$celltype=factor(plotdf$celltype,levels = unique(as.character(sce.markers$cluster)))
plotdf$gene=factor(plotdf$gene,levels = rev(as.character(Top5)))
plotdf$exp=ifelse(plotdf$exp>3,3,plotdf$exp)
sc_marker_dotplot1<-plotdf%>%ggplot(aes(x=celltype,y=gene,size=ratio,color=exp))+geom_poin
t()+
  scale_x_discrete("")+scale_y_discrete("")+
  scale_color_gradientn(colours = rev(c("#FFD92F","#FEE391",brewer.pal(11,
"Spectral")[7:11])))+
```

```
  scale_size_continuous(limits = c(0,1))+theme_bw()+
  theme(
    axis.text.x.bottom = element_text(hjust = 1, vjust = 1, angle = 45)
  )
sc_marker_dotplot1
ggsave('results/sc_marker_dotplot1.pdf',sc_marker_dotplot1,height = 7,width = 9)

#绘图

mycolor =ggsci::pal_jama()(9)

fig1a = DimPlot(sce,group.by = 'Samples',
                reduction="umap",
                label = "F",
                pt.size = 0.5,
                label.size = 5) +
  theme(axis.line = element_line(size=0.1, colour = "black"),
        #axis.text = element_blank(),
        #axis.title = element_blank(),
        axis.ticks = element_blank()
  ) +ggtitle('')+guides(colour = guide_legend(ncol = 1))

fig1a

fig1b<-DimPlot(sce,cols=mycolor,group.by = 'seurat_clusters',
               reduction="umap",split.by = 'type',
               label = "F",
               pt.size = 0.5,
               label.size = 5) +
  theme(axis.line = element_line(size=0.1, colour = "black"),
        #axis.text = element_blank(),
        #axis.title = element_blank(),
        axis.ticks = element_blank()
  ) +ggtitle('')
fig1b
Idents(sce)='seurat_clusters'
library("ggplot2")
sample_clust<-as.matrix(table(sce$Samples,sce$seurat_clusters))
sample_clust=apply(sample_clust,1,function(x){return(x/sum(x))})
sample_clust=reshape2::melt(sample_clust)
colnames(sample_clust)<-c("cluster","Samples","proportion")
sample_clust$cluster=paste0('CAF_',sample_clust$cluster)
write.table(sample_clust,'results/sample_clust1.txt',quote = F,row.names = T,sep='\t')
```

```
clust_freq<-as.data.frame(table(sce$Samples))
colnames(clust_freq)=c('Samples','cell_num')
clust_freq=clust_freq[order(clust_freq$cell_num,decreasing = T),]
clust_freq$Samples=factor(clust_freq$Samples,levels = clust_freq$Samples)
sample_clust$Samples=factor(sample_clust$Samples,levels =clust_freq$Samples)

fig1e1<-ggplot(sample_clust,aes(x = Samples,y = proportion,fill=cluster))+
  geom_bar(stat = "identity", position = "fill") +
  ggtitle("") +scale_fill_manual(values = mycolor[1:5])+
  theme_bw() +
  theme(axis.ticks.length = unit(0.1, 'cm'),
        legend.position = "left") +xlab('')+
  coord_flip()+scale_y_continuous(expand = expand_scale(mult = c(0, 0)))
fig1e1

fig1e2<-ggplot(clust_freq,aes(x = Samples,y = cell_num,fill=Samples))+
  geom_bar(stat="identity")+ggtitle("") +
  theme_bw() + scale_fill_manual(values = sample_color)+
  theme(axis.ticks.length = unit(0, 'cm'),
        axis.text.y = element_blank(),
        axis.title.y = element_blank()) +coord_flip()+
  scale_y_continuous(expand = expand_scale(mult = c(0,
0)))+ylim(0,max(clust_freq$cell_num)+10)
fig1e2

fig1e3<-ggpubr::ggarrange(fig1e1,fig1e2,nrow = 1,ncol = 2,widths = c(2,1))
fig1e3
library(clusterProfiler)
library(org.Hs.eg.db)
ids=bitr(sce.markers$gene,'SYMBOL','ENTREZID','org.Hs.eg.db') ## 将 SYMBOL 转成 ENTREZID
sce.markers2=merge(sce.markers,ids,by.x='gene',by.y='SYMBOL')
gcSample=split(sce.markers2$ENTREZID, sce.markers2$cluster)
## KEGG
sce.markers2.enrich.res <- compareCluster(gcSample,
                                          fun = "enrichKEGG",
                                          organism = "hsa", pvalueCutoff = 0.05)
fig1f<-dotplot(sce.markers2.enrich.res)+
  theme(axis.text.x = element_text(angle = 45,hjust = 1,size=10),
        axis.text.y=element_text(size=10))
fig1f

save(sce,file = 'sce.RData')
load('sce.RData')
library(copykat)
```

```
copykat <- function (rawmat = rawdata, id.type = "S", cell.line = "no",
                                  ngene.chr = 0, LOW.DR = 0.05, UP.DR = 0.1, win.size = 25,
                                  norm.cell.names  =  "",  KS.cut  =  0.1,  sam.name  =  "",  distance  =
"euclidean",
                                  n.cores = 1) {
    start_time <- Sys.time()
    set.seed(1)
    sample.name <- paste(sam.name, "_copykat_", sep = "")
    print("running copykat v1.0.4")
    print("step1: read and filter data ...")
    print(paste(nrow(rawmat), " genes, ", ncol(rawmat), " cells in raw data",
                      sep = ""))
    # genes.raw <- apply(rawmat, 2, function(x) (sum(x > 0)))
    # if (sum(genes.raw > 200) == 0)
    #      stop("none cells have more than 200 genes")
    # if (sum(genes.raw < 100) > 1) {
    #      rawmat <- rawmat[, -which(genes.raw < 200)]
    #      print(paste("filtered out ", sum(genes.raw <= 200),
    #                              " cells with less than 200 genes; remaining ", ncol(rawmat),
    #                              " cells", sep = ""))
    # }
    der <- apply(rawmat, 1, function(x) (sum(x > 0)))/ncol(rawmat)
    if (sum(der > LOW.DR) >= 1) {
        rawmat <- rawmat[which(der > LOW.DR), ]
        print(paste(nrow(rawmat), " genes past LOW.DR filtering",
                          sep = ""))
    }
    WNS1 <- "data quality is ok"
    if (nrow(rawmat) < 7000) {
        WNS1 <- "low data quality"
        UP.DR <- LOW.DR
        print("WARNING: low data quality; assigned LOW.DR to UP.DR...")
    }
    print("step 2: annotations gene coordinates ...")
    anno.mat <- annotateGenes.hg20(mat = rawmat, ID.type = id.type)
    anno.mat <- anno.mat[order(anno.mat$abspos, decreasing = FALSE),
    ]
    HLAs <- anno.mat$hgnc_symbol[grep("^HLA-", anno.mat$hgnc_symbol)]
    toRev <- which(anno.mat$hgnc_symbol %in% c(as.vector(cyclegenes[[1]]), HLAs))
    # if (length(toRev) > 0) {
    #      anno.mat <- anno.mat[-toRev, ]
    # }
    # ToRemov2 <- NULL
    # for (i in 8:ncol(anno.mat)) {
```

```
#     cell <- cbind(anno.mat$chromosome_name, anno.mat[, i])
#     cell <- cell[cell[, 2] != 0, ]
#     if (length(as.numeric(cell)) < 5) {
#         rm <- colnames(anno.mat)[i]
#         ToRemov2 <- c(ToRemov2, rm)
#     }
#     else if (length(rle(cell[, 1])$length) < 23 | min(rle(cell[,
#                                                                1])$length) <
ngene.chr) {
#         rm <- colnames(anno.mat)[i]
#         ToRemov2 <- c(ToRemov2, rm)
#     }
#     i <- i + 1
# }
# if (length(ToRemov2) == (ncol(anno.mat) - 7))
#     stop("all cells are filtered")
# if (length(ToRemov2) > 0) {
#     anno.mat <- anno.mat[, -which(colnames(anno.mat) %in%
#                                       ToRemov2)]
# }
rawmat3 <- data.matrix(anno.mat[, 8:ncol(anno.mat)])
norm.mat <- log(sqrt(rawmat3) + sqrt(rawmat3 + 1))
norm.mat <- apply(norm.mat, 2, function(x) (x <- x - mean(x)))
colnames(norm.mat) <- colnames(rawmat3)
print("step 3: smoothing data with dlm ...")
dlm.sm <- function(c) {
    model <- dlm::dlmModPoly(order = 1, dV = 0.16, dW = 0.001)
    x <- dlm::dlmSmooth(norm.mat[, c], model)$s
    x <- x[2:length(x)]
    x <- x - mean(x)
}
test.mc <- parallel::mclapply(1:ncol(norm.mat), dlm.sm,
                              mc.cores = n.cores)
norm.mat.smooth <- matrix(unlist(test.mc), ncol = ncol(norm.mat),
                          byrow = FALSE)
colnames(norm.mat.smooth) <- colnames(norm.mat)
print("step 4: measuring baselines ...")
if (cell.line == "yes") {
    print("running pure cell line mode")
    relt <- baseline.synthetic(norm.mat = norm.mat.smooth,
                               min.cells = 10, n.cores = n.cores)
    norm.mat.relat <- relt$expr.relat
    CL <- relt$cl
    WNS <- "run with cell line mode"
```

```
        preN <- NULL
    }
    else if (length(norm.cell.names) > 1) {
        NNN <- length(colnames(norm.mat.smooth)[which(colnames(norm.mat.smooth) %in%
                                                            norm.cell.names)])
        print(paste(NNN, " known normal cells found in dataset",
                    sep = ""))
        if (NNN == 0)
            stop("known normal cells provided; however none existing in testing dataset")
        print("run with known normal...")
        basel <- apply(norm.mat.smooth[, which(colnames(norm.mat.smooth) %in%
                                                     norm.cell.names)], 1, median)
        print("baseline is from known input")
        d <- parallelDist::parDist(t(norm.mat.smooth), threads = n.cores,
                                   method = "euclidean")
        km <- 6
        fit <- hclust(d, method = "ward.D2")
        CL <- cutree(fit, km)
        while (!all(table(CL) > 5)) {
            km <- km - 1
            CL <- cutree(fit, k = km)
            if (km == 2) {
                break
            }
        }
        WNS <- "run with known normal"
        preN <- norm.cell.names
        norm.mat.relat <- norm.mat.smooth - basel
    }
    else {
        basa <- baseline.norm.cl(norm.mat.smooth = norm.mat.smooth,
                                 min.cells = 5, n.cores = n.cores)
        basel <- basa$basel
        WNS <- basa$WNS
        preN <- basa$preN
        CL <- basa$cl
        if (WNS == "unclassified.prediction") {
            Tc <- colnames(rawmat)[which(as.numeric(apply(rawmat[which(rownames(rawmat) %in%
c("PTPRC", "LYZ", "PECAM1")), ], 2, mean)) > 1)]
            length(Tc)
            preN <- intersect(Tc, colnames(norm.mat.smooth))
            if (length(preN) > 5) {
                print("start manual mode")
                WNS <- paste("copykat failed in locating normal cells; manual adjust performed with ",
```

```
                            length(preN), " immune cells", sep = "")
            print(WNS)
            basel <- apply(norm.mat.smooth[, which(colnames(norm.mat.smooth) %in%
                                                            preN)], 1, mean)
        }else {
            basa <- baseline.GMM(CNA.mat = norm.mat.smooth,
                                    max.normal = 5, mu.cut = 0.05, Nfraq.cut = 0.99,
                                    RE.before = basa, n.cores = n.cores)
            basel <- basa$basel
            WNS <- basa$WNS
            preN <- basa$preN
        }
    }
    norm.mat.relat <- norm.mat.smooth - basel
  }
  DR2 <- apply(rawmat3, 1, function(x) (sum(x > 0)))/ncol(rawmat3)
  norm.mat.relat <- norm.mat.relat[which(DR2 >= UP.DR), ]
  anno.mat2 <- anno.mat[which(DR2 >= UP.DR), ]
  # ToRemov3 <- NULL
  # for (i in 8:ncol(anno.mat2)) {
  #     cell <- cbind(anno.mat2$chromosome_name, anno.mat2[,
  #                                                          i])
  #     cell <- cell[cell[, 2] != 0, ]
  #     if (length(as.numeric(cell)) < 5) {
  #         rm <- colnames(anno.mat2)[i]
  #         ToRemov3 <- c(ToRemov3, rm)
  #     }
  #     else if (length(rle(cell[, 1])$length) < 23 | min(rle(cell[,
  #                                                                1])$length) <
ngene.chr) {
  #         rm <- colnames(anno.mat2)[i]
  #         ToRemov3 <- c(ToRemov3, rm)
  #     }
  #     i <- i + 1
  # }
  # if (length(ToRemov3) == ncol(norm.mat.relat))
  #     stop("all cells are filtered")
  # if (length(ToRemov3) > 0) {
  #     norm.mat.relat <- norm.mat.relat[, -which(colnames(norm.mat.relat) %in%
  #                                                       ToRemov3)]
  # }
  CL <- CL[which(names(CL) %in% colnames(norm.mat.relat))]
  CL <- CL[order(match(names(CL), colnames(norm.mat.relat)))]
  print("step 5: segmentation...")
```

```
    results <- CNA.MCMC(clu = CL, fttmat = norm.mat.relat, bins = win.size,
                                      cut.cor = KS.cut, n.cores = n.cores)
    if (length(results$breaks) < 25) {
        print("too few breakpoints detected; decreased KS.cut to 50%")
        results <- CNA.MCMC(clu = CL, fttmat = norm.mat.relat,
                                          bins = win.size, cut.cor = 0.5 * KS.cut, n.cores = n.cores)
    }
    if (length(results$breaks) < 25) {
        print("too few breakpoints detected; decreased KS.cut to 75%")
        results <- CNA.MCMC(clu = CL, fttmat = norm.mat.relat,
                                          bins = win.size, cut.cor = 0.5 * 0.5 * KS.cut, n.cores = n.cores)
    }
    if (length(results$breaks) < 25)
        stop("too few segments; try to decrease KS.cut; or improve data")
    colnames(results$logCNA) <- colnames(norm.mat.relat)
    results.com <- apply(results$logCNA, 2, function(x) (x <- x -
                                                                                mean(x)))
    RNA.copycat <- cbind(anno.mat2[, 1:7], results.com)
    write.table(RNA.copycat, paste(sample.name, "CNA_raw_results_gene_by_cell.txt",
                                                   sep = ""), sep = "\t", row.names = FALSE, quote = F)
    print("step 6: convert to genomic bins...")
    Aj <- convert.all.bins.hg20(DNA.mat = DNA.hg20, RNA.mat = RNA.copycat,
                                                n.cores = n.cores)
    uber.mat.adj <- data.matrix(Aj$RNA.adj[, 4:ncol(Aj$RNA.adj)])
    print("step 7: adjust baseline ...")
    if (cell.line == "yes") {
        mat.adj <- data.matrix(Aj$RNA.adj[, 4:ncol(Aj$RNA.adj)])
        write.table(cbind(Aj$RNA.adj[, 1:3], mat.adj), paste(sample.name, "CNA_results.txt", sep =
""),
                          sep = "\t", row.names = FALSE, quote = F)
        if (distance == "euclidean") {
            hcc <- hclust(parallelDist::parDist(t(mat.adj),threads = n.cores, method = distance),
method = "ward.D")
        }else {
            hcc <- hclust(as.dist(1 - cor(mat.adj, method = distance)), method = "ward.D")
        }
        saveRDS(hcc, file = paste(sample.name, "clustering_results.rds", sep = ""))
        print("step 8: ploting heatmap ...")
        my_palette <- colorRampPalette(rev(RColorBrewer::brewer.pal(n = 3, name = "RdBu")))(n =
999)
        chr <- as.numeric(Aj$DNA.adj$chrom)%%2 + 1
        rbPal1 <- colorRampPalette(c("black", "grey"))
        CHR <- rbPal1(2)[as.numeric(chr)]
        chr1 <- cbind(CHR, CHR)
```

```
    if (ncol(mat.adj) < 3000) { h <- 10}else {h <- 15}
    col_breaks = c(seq(-1, -0.4, length = 50), seq(-0.4, -0.2, length = 150), seq(-0.2, 0.2, length = 
600),
                   seq(0.2, 0.4, length = 150), seq(0.4, 1, length = 50))
    if (distance == "euclidean") {
      jpeg(paste(sample.name, "heatmap.jpeg", sep = ""),
           height = h * 250, width = 4000, res = 100)
      heatmap.3(t(mat.adj), dendrogram = "r", distfun = function(x) 
parallelDist::parDist(x,threads = n.cores, method = distance), hclustfun = function(x) 
hclust(x,method = "ward.D"), ColSideColors = chr1, Colv = NA,
                Rowv = TRUE, notecol = "black", col = my_palette,
                breaks = col_breaks, key = TRUE, keysize = 1,
                density.info = "none", trace = "none", cexRow = 0.1,
                cexCol = 0.1, cex.main = 1, cex.lab = 0.1, symm = F,
                symkey = F, symbreaks = T, cex = 1, main = paste(WNS1, "; ", WNS, sep = ""), 
cex.main = 4, margins = c(10, 10))
      dev.off()
    }
    else {
      jpeg(paste(sample.name, "heatmap.jpeg", sep = ""),
           height = h * 250, width = 4000, res = 100)
      heatmap.3(t(mat.adj), dendrogram = "r", distfun = function(x) as.dist(1 -cor(t(x), method = 
distance)), hclustfun = function(x) hclust(x,  method = "ward.D"), ColSideColors = chr1, Colv = 
NA,
                Rowv = TRUE, notecol = "black", col = my_palette,
                breaks = col_breaks, key = TRUE, keysize = 1,
                density.info = "none", trace = "none", cexRow = 0.1,
                cexCol = 0.1, cex.main = 1, cex.lab = 0.1, symm = F,
                symkey = F, symbreaks = T, cex = 1, main = paste(WNS1, "; ", WNS, sep = ""), 
cex.main = 4, margins = c(10,10))
      dev.off()
    }
    end_time <- Sys.time()
    print(end_time - start_time)
    reslts <- list(cbind(Aj$RNA.adj[, 1:3], mat.adj), hcc)
    names(reslts) <- c("CNAmat", "hclustering")
    return(reslts)
  }
  else {
    if (distance == "euclidean") {
      hcc <- hclust(parallelDist::parDist(t(uber.mat.adj), threads = n.cores, method = distance), 
method = "ward.D")
    }
    else {
```

```
    hcc <- hclust(as.dist(1 - cor(uber.mat.adj, method = distance)), method = "ward.D")
}
hc.umap <- cutree(hcc, 2)
names(hc.umap) <- colnames(results.com)
cl.ID <- NULL
for (i in 1:max(hc.umap)) {
    cli <- names(hc.umap)[which(hc.umap == i)]
    pid <- length(intersect(cli, preN))/length(cli)
    cl.ID <- c(cl.ID, pid)
    i <- i + 1
}
com.pred <- names(hc.umap)
com.pred[which(hc.umap == which(cl.ID == max(cl.ID)))] <- "diploid"
com.pred[which(hc.umap == which(cl.ID == min(cl.ID)))] <- "nondiploid"
names(com.pred) <- names(hc.umap)
results.com.rat <- uber.mat.adj - apply(uber.mat.adj[, which(com.pred == "diploid")], 1,
mean)
results.com.rat <- apply(results.com.rat, 2, function(x) (x <- x-mean(x)))
results.com.rat.norm <- results.com.rat[, which(com.pred == "diploid")]
dim(results.com.rat.norm)
cf.h <- apply(results.com.rat.norm, 1, sd)
base <- apply(results.com.rat.norm, 1, mean)
adjN <- function(j) {a <- results.com.rat[, j]
a[abs(a - base) <= 0.25 * cf.h] <- mean(a)
a
}
mc.adjN <- parallel::mclapply(1:ncol(results.com.rat),    adjN, mc.cores = n.cores)
adj.results <- matrix(unlist(mc.adjN), ncol = ncol(results.com.rat), byrow = FALSE)
colnames(adj.results) <- colnames(results.com.rat)
rang <- 0.5 * (max(adj.results) - min(adj.results))
mat.adj <- adj.results/rang
print("step 8: final prediction ...")
if (distance == "euclidean") {
    hcc <- hclust(parallelDist::parDist(t(mat.adj), threads = n.cores, method = distance),
                            method = "ward.D")
}
else {
    hcc <- hclust(as.dist(1 - cor(mat.adj, method = distance)), method = "ward.D")
}
hc.umap <- cutree(hcc, 2)
names(hc.umap) <- colnames(results.com)
saveRDS(hcc, file = paste(sample.name, "clustering_results.rds",
                                        sep = ""))
cl.ID <- NULL
```

```
        for (i in 1:max(hc.umap)) {
            cli <- names(hc.umap)[which(hc.umap == i)]
            pid <- length(intersect(cli, preN))/length(cli)
            cl.ID <- c(cl.ID, pid)
            i <- i + 1
        }
        com.preN <- names(hc.umap)
        com.preN[which(hc.umap == which(cl.ID == max(cl.ID)))] <- "diploid"
        com.preN[which(hc.umap == which(cl.ID == min(cl.ID)))] <- "aneuploid"
        names(com.preN) <- names(hc.umap)
        if (WNS == "unclassified.prediction") {
            com.preN[which(com.preN == "diploid")] <- "c1:diploid:low.conf"
            com.preN[which(com.preN == "nondiploid")] <- "c2:aneuploid:low.conf"
        }
        print("step 9: saving results...")
        res <- cbind(names(com.preN), com.preN)
        colnames(res) <- c("cell.names", "copykat.pred")
        write.table(res, paste(sample.name, "prediction.txt",
                                            sep = ""), sep = "\t", row.names = FALSE, quote = FALSE)
        write.table(cbind(Aj$RNA.adj[, 1:3], mat.adj), paste(sample.name,"CNA_results.txt", sep =
""), sep = "\t", row.names = FALSE, quote = F)
        print("step 10: ploting heatmap ...")
        my_palette <- colorRampPalette(rev(RColorBrewer::brewer.pal(n = 3, name = "RdBu")))(n =
999)
        chr <- as.numeric(Aj$DNA.adj$chrom)%%2 + 1
        rbPal1 <- colorRampPalette(c("black", "grey"))
        CHR <- rbPal1(2)[as.numeric(chr)]
        chr1 <- cbind(CHR, CHR)
        rbPal5 <- colorRampPalette(RColorBrewer::brewer.pal(n = 8, name = "Dark2")[2:1])
        compreN_pred <- rbPal5(2)[as.numeric(factor(com.preN))]
        cells <- rbind(compreN_pred, compreN_pred)
        if (ncol(mat.adj) < 3000) {
            h <- 10
        }
        else {
            h <- 15
        }
        col_breaks = c(seq(-1, -0.4, length = 50), seq(-0.4,
                                                                -0.2, length = 150), seq(-0.2, 0.2,
length = 600),
                            seq(0.2, 0.4, length = 150), seq(0.4, 1, length = 50))
        if (distance == "euclidean") {
            jpeg(paste(sample.name, "heatmap.jpeg", sep = ""),
                    height = h * 250, width = 4000, res = 100)
```

```
        heatmap.3(t(mat.adj), dendrogram = "r", distfun = function(x) parallelDist::parDist(x,
threads = n.cores, method = distance), hclustfun = function(x) hclust(x,method = "ward.D"),
ColSideColors = chr1, RowSideColors = cells,    Colv = NA, Rowv = TRUE, notecol = "black", col =
my_palette,    breaks = col_breaks, key = TRUE, keysize = 1, ensity.info = "none", trace = "none",
cexRow = 0.1, cexCol = 0.1, cex.main = 1, cex.lab = 0.1, symm = F, symkey = F, symbreaks = T, cex =
1, main = paste(WNS1, "; ", WNS, sep = ""), cex.main = 4, margins = c(10,10))
        legend("topright", paste("pred.", names(table(com.preN)), sep = ""), pch = 15, col =
RColorBrewer::brewer.pal(n = 8, name = "Dark2")[2:1], cex = 1)
        dev.off()
      }
      else {
        jpeg(paste(sample.name, "heatmap.jpeg", sep = ""),
              height = h * 250, width = 4000, res = 100)
        heatmap.3(t(mat.adj), dendrogram = "r", distfun = function(x) as.dist(1 -

cor(t(x), method = distance)), hclustfun = function(x) hclust(x,method = "ward.D"), ColSideColors
= chr1, RowSideColors = cells,
                    Colv = NA, Rowv = TRUE, notecol = "black", col = my_palette,
                    breaks = col_breaks, key = TRUE, keysize = 1,
                    density.info = "none", trace = "none", cexRow = 0.1,
                    cexCol = 0.1, cex.main = 1, cex.lab = 0.1, symm = F,
                    symkey = F, symbreaks = T, cex = 1, main = paste(WNS1, "; ", WNS, sep = ""),
                    cex.main = 4, margins = c(10, 10))
        legend("topright", paste("pred.", names(table(com.preN)),sep = ""),
                pch = 15, col = RColorBrewer::brewer.pal(n = 8, name = "Dark2")[2:1], cex = 1)
        dev.off()
      }
      end_time <- Sys.time()
      print(end_time - start_time)
      reslts <- list(res, cbind(Aj$RNA.adj[, 1:3], mat.adj),
                      hcc)
      names(reslts) <- c("prediction", "CNAmat", "hclustering")
      return(reslts)
    }
}
copykat.test <- copykat(rawmat=sce@assays$RNA@counts,
                        id.type="S",
                        cell.line="no",
                        ngene.chr=5,
                        #每个染色体中至少有 5 个基因来计算 DNA 拷贝数
                        win.size=25,
                        #每个片段至少取 25 个基因
                        KS.cut=0.15,
                        #0-1,值越大灵敏度越低
```

```
                                        sam.name="LUAD",
                                        #随意固定一个名称
                                        distance="euclidean",
                                        n.cores=1
                                        #并行计算
)
save(copykat.test,file = 'copykat.test.RData')

copykat.test<-read.delim('LUAD_copykat_prediction.txt',sep='\t',header = T)
head(copykat.test)
table(copykat.test$copykat.pred)
rownames(copykat.test)=copykat.test$cell.names
copykat.test=copykat.test[rownames(sce@meta.data),]
sce <- AddMetaData(sce, copykat.test$copykat.pred,col.name = "copykat.pred")
sce$copykat.pred[is.na(sce$copykat.pred)]<-'Unknown'
table(sce$copykat.pred)
# aneuploid      diploid
#347             241
sce$copykat.pred=ifelse(sce$copykat.pred=='aneuploid','malignant','no_malignant')

save(sce,file = 'sce.RData')
fig1h<-DimPlot(sce,cols=c('red','blue'),group.by = 'copykat.pred',
                        reduction="umap",
                        label = "F",
                        pt.size = 0.5,
                        label.size = 5) +
  theme(axis.line = element_line(size=0.1, colour = "black"),
          #axis.text = element_blank(),
          #axis.title = element_blank(),
          axis.ticks = element_blank()
  ) +ggtitle('')
fig1h

fig1ef<-ggarrange(fig1e3,fig1f,fig1h,labels = c('D','E','F'),nrow = 1,ncol = 3,widths = c(1.2,1.3,1))

fig1ab<-ggarrange(fig1a,fig1b,nrow = 1,ncol=2,labels = c('A','B'),widths = c(1,1.5))
fig1=ggarrange(fig1ab,sc_marker_dotplot,fig1ef,labels = c('','C',''),nrow = 3,ncol = 1,heights =
c(2,1,1))

ggsave(filename = 'results/Fig1.pdf',plot = fig1,he=15,wi=18)
ggsave(filename = 'results/Fig1.jpg',plot = fig1,he=15,wi=18)
```

```
library(Seurat)
library(dplyr)
library(ggplot2)
library(magrittr)
library(gtools)
library(stringr)
library(Matrix)
library(tidyverse)
library(patchwork)
library(data.table)
library(RColorBrewer)
library(ggpubr)
library(ggsci)
load('../01.scRNA/sce.RData')
pathway.score<-function(exp,gene){
  ssGSEAScore_by_genes<-function(gene.exp,genes){
    gs=GSEABase::GeneSet(setName='GeneSet',
setIdentifier=paste0("101"),geneIds=unique(genes),GSEABase::SymbolIdentifier())

    gsc <- GSEABase::GeneSetCollection(list(gs))
    fl <- tempfile()
    GSEABase::toGmt(gsc, fl)
    cgeneset=GSEABase::getGmt(fl)
    ssGSEA.geneset <- GSVA::gsva(as.matrix(gene.exp), cgeneset,method='ssgsea',
                                             min.sz=1, max.sz=5000, verbose=TRUE)
    return(ssGSEA.geneset)
  }

  pathway_score<-data.frame()
  for (i in unique(gene[,2])){
    gene_set=gene[gene[,2]==i,1]
    score=ssGSEAScore_by_genes(exp,gene_set)
    rownames(score)=i
    pathway_score=rbind.data.frame(pathway_score,score)
  }
  return(t(pathway_score))
}
#_pmid_29625050
tumor.pathway=read.delim('pmid_29625050_pathway.txt',sep='\t',header = T)
head(tumor.pathway)
tumor.pathway=tumor.pathway[,c("Gene","OG.TSG")]

#每一个细胞计算得分
```

```
tumor.pathway.score<-pathway.score(exp = as.matrix(sce@assays$RNA@counts),gene = tumor.pathway)
head(tumor.pathway.score)

tumor.pathway.score.group<-merge(data.frame(cell.names=rownames(sce@meta.data),
                                            sce@meta.data),

data.frame(cell.names=rownames(tumor.pathway.score),
                                            tumor.pathway.score),
                                  by='cell.names')
rownames(tumor.pathway.score.group)=tumor.pathway.score.group$cell.names
head(tumor.pathway.score.group)
tumor.pathway.score.group=tumor.pathway.score.group[,-1]




copykat.test=data.frame(cell.names=rownames(sce@meta.data),
                        copykat.pred=sce@meta.data$copykat.pred)

head(copykat.test)

table(copykat.test$copykat.pred)
tumor.score.copy<-cbind.data.frame(tumor.pathway.score.group[copykat.test$cell.names,],
                                   copykat.pred=copykat.test$copykat.pred)
head(tumor.score.copy)
table(tumor.score.copy$copykat.pred)
tumor.score.copy$seurat_clusters=paste0('CAF_',tumor.score.copy$seurat_clusters)
library(pheatmap)
head(tumor.score.copy)
table(tumor.score.copy$copykat.pred)

tumor.score.copy=tumor.score.copy[,c("Samples",'seurat_clusters',

'CellCyle','HIPPO','MYC','NOTCH','NRF1','PI3K','TGF.Beta','RAS','TP53','WNT',
                                     'copykat.pred')]
colnames(tumor.score.copy)[9]
colnames(tumor.score.copy)[9]='TGF-Beta'
mat=tumor.score.copy[,as.character(unique(tumor.pathway$OG.TSG))]
anno_col<-tumor.score.copy[,c("seurat_clusters","copykat.pred")]
anno_col=anno_col[order(anno_col$copykat.pred,anno_col$seurat_clusters),]
pdf('results/Fig2a.pdf',height = 9,width = 12)
pheatmap::pheatmap(t(mat[rownames(anno_col),]),scale = 'row',
                   show_colnames = F,annotation_col = anno_col,
```

```
                                        cluster_cols = F,cluster_rows =T,
                                        color = colorRampPalette(c("blue", "white","red"))(100),
                                        annotation_names_row = F,
                                        annotation_colors                                          =
list(copykat.pred=c('malignant'='red','no_malignant'='blue')),
                                        breaks = unique(c(seq(-2, 2, length=100))))
dev.off()
write.table(tumor.score.copy,'results/tumor.score.copy.txt',quote = F,row.names = T,sep='\t')
clust.malig<-table(tumor.score.copy$copykat.pred,tumor.score.copy$seurat_clusters)
clust.malig
write.table(clust.malig,'results/clust.malig.txt',quote = F,sep='\t',row.names = T)

plotiBarplot<-function(dat,palette,ist=F,margin=T,lineCol='black',legTitle='Group',showValue=F,sh
owLine=T){
   library(ggplot2)
   xlb='';ylb='';lineW=0.5;xangle=0;isAuto=T;
   #library(tidyverse)
   #library(reshape2)
   #library(optparse)
   if(ist){
      dat=t(dat)
   }
   lbc=colnames(dat)
   lbr=row.names(dat)
   bk_dat=dat
   if(margin){
      dat=dat%*%diag(1/c(apply(t(dat), 1, sum)))
   }
   row.names(dat)=paste0('R',1:(nrow(dat)))
   colnames(dat)=paste0('C',1:(ncol(dat)))
   row.names(bk_dat)=paste0('R',1:(nrow(bk_dat)))
   colnames(bk_dat)=paste0('C',1:(ncol(bk_dat)))
   #df=cbind(bg=paste0('R',1:nrow(dat)),dat)
   #colnames(df)=c('bg',paste0('C',1:(ncol(dat))))
   tp.dat=as.data.frame(cbind(bg=row.names(dat),dat))
   tp.dat[,1]=as.character(tp.dat[,1])
   for(i in 2:ncol(tp.dat)){
      tp.dat[,i]=as.numeric(as.character(tp.dat[,i]))
   }
   mt.df=reshape2::melt(tp.dat)
   colnames(mt.df)=c('bg','variable','value')

   pg=ggplot(mt.df, aes(x=variable, y=value, fill=bg))+
      geom_bar(stat = "identity", width=lineW, col=lineCol)
```

```
  if(showLine){
    for (i in 2:(ncol(tp.dat)-1)) {
      tmp=tp.dat[order(tp.dat[,1],decreasing = T),]
      tmp[,i]=base::cumsum(tmp[,i])
      tmp[,i+1]=base::cumsum(tmp[,i+1])
      colnames(tmp)[c(i,i+1)]=c('STY','ED')
      tmp1=cbind(tmp,STX=rep(i-1+lineW/2,nrow(tmp))
                 ,EDX=rep(i-lineW/2,nrow(tmp)))
      pg=pg+geom_segment(data=tmp1,aes(x=STX, xend=EDX, y=STY, yend=ED))
    }
  }

  if(showValue){
    pg=pg+geom_text(data=mt.df,aes(label=sprintf("%0.2f", round(value, digits = 2))),position=position_stack(vjust=0.5))
  }
  pg=pg+scale_x_discrete(breaks = paste0('C',1:(ncol(dat))),label = lbc)
  pg=pg+labs(x=xlb, y=ylb)+theme(legend.position = "bottom")
  #pg=pg+scale_fill_discrete(breaks = paste0('R',1:nrow(dat)),label = lbr,name=legTitle)
  pg=pg+scale_fill_manual(breaks = paste0('R',1:nrow(dat)),label = lbr,name=legTitle,values=palette)
  if(xangle>0){
    pg=pg+theme(axis.text.x = element_text(angle = xangle, hjust = 1),legend.position = "bottom")
  }

  g.tb=matrix(0,nrow=ncol(dat),ncol=ncol(dat))
  for(i in 1:(ncol(dat))){
    for(j in 1:ncol(dat)){
      if(i!=j){
        g.tb[i,j]=round(-log10((chisq.test(bk_dat[,c(i,j)])$p.value)),2)
      }
    }
  }
  colnames(g.tb)=lbc
  row.names(g.tb)=lbc
  g.tb=reshape2::melt(g.tb)
  colnames(g.tb)=c('A1','A2','A3')
  g.tb$A4=paste0(g.tb[,3],ifelse(g.tb[,3]>-log10(0.05),'(*)',''))
  stable.p=ggplot(g.tb, aes(A1, A2)) + geom_tile(aes(fill = A3),colour = "white") +xlab('')+ylab('')+ scale_fill_gradient(low = "white",high = "steelblue")+geom_text(aes(x=A1,y=A2,label=A4))+theme(legend.position="none",axis.title.x=element_blank(),axis.text.x=element_blank())
  stable.p=stable.p+ggtitle('-log10(anova p value)')
```

```
  if(isAuto){
    g1=ggpubr::ggarrange(stable.p,pg, ncol = 1, nrow = 2,heights = c(0.5,1),align = "hv")
    return(g1)
  }else{
    return(list(Bar=pg,Table=stable.p))
  }
}
#cellcolor =ggsci::pal_jama()(9)

fig2b<-plotiBarplot(dat = clust.malig,palette=c('red','blue'),ist = F,margin=T,lineCol='black',
                    legTitle = 'Predict',showValue=T,showLine=T)
fig2b
ggsave('results/Fig2b.pdf',fig2b,height = 7,width = 10)
head(tumor.pathway.score.group)
table(tumor.score.copy$seurat_clusters)

#C0
tumor.pathway.score.group1=tumor.score.copy[tumor.score.copy$seurat_clusters=='CAF_0',]
#箱线图
Muti_Boxplot<-function(dat,group,group_cols,leg,
                       test_method = 'wilcox.test',ylabs){
  library(ggpubr)
  library(reshape2)
  dat1=reshape2::melt(cbind.data.frame(dat,group))
  p=ggboxplot(dat1, x='variable', y='value',
              fill = "group", color = "black",
              palette = group_cols,
              ylab=ylabs,xlab='',
              add = "boxplot")+
    stat_compare_means(aes(group=group),method = test_method,
                       symnum.args=list(cutpoints = c(0, 0.001, 0.01, 0.05, 1),
                                        symbols = c("***", "**", "*", "ns")),
                       label = "p.signif")+
    theme(axis.text.x = element_text(angle = 30,hjust = 1))+labs(fill=leg)
  return(p)
}


fig2d<-Muti_Boxplot(dat
=tumor.pathway.score.group1[,as.character(unique(tumor.pathway$OG.TSG))],
                    group = tumor.pathway.score.group1$copykat.pred,
                    group_cols = ggsci::pal_lancet()(9)[c(2,1)],
                    test_method = 'wilcox.test',
                    leg = 'CAF_0',ylab = 'GSVA Score')
```

```
fig2d
ggsave('results/Fig2c.pdf',fig2d,height = 5,width = 9)

#C1
tumor.pathway.score.group2=tumor.score.copy[tumor.score.copy$seurat_clusters=='CAF_1',]
fig2e<-Muti_Boxplot(dat
=tumor.pathway.score.group2[,as.character(unique(tumor.pathway$OG.TSG))],
                        group = tumor.pathway.score.group2$copykat.pred,
                        group_cols = ggsci::pal_lancet()(9)[c(2,1)],
                        test_method = 'wilcox.test',
                        leg = 'CAF_1',ylab = 'GSVA Score')
fig2e
ggsave('results/Fig2d.pdf',fig2e,height = 5,width = 9)

#C2
tumor.pathway.score.group3=tumor.score.copy[tumor.score.copy$seurat_clusters=='CAF_2',]
fig2f<-Muti_Boxplot(dat
=tumor.pathway.score.group3[,as.character(unique(tumor.pathway$OG.TSG))],
                        group = tumor.pathway.score.group3$copykat.pred,
                        group_cols = ggsci::pal_lancet()(9)[c(2,1)],
                        test_method = 'wilcox.test',
                        leg = 'CAF_2',ylab = 'GSVA Score')
fig2f
ggsave('results/Fig2e.pdf',fig2f,height = 5,width = 9)

#C3
tumor.pathway.score.group4=tumor.score.copy[tumor.score.copy$seurat_clusters=='CAF_3',]
fig2g<-Muti_Boxplot(dat
=tumor.pathway.score.group4[,as.character(unique(tumor.pathway$OG.TSG))],
                        group = tumor.pathway.score.group4$copykat.pred,
                        group_cols = ggsci::pal_lancet()(9)[c(2,1)],
                        test_method = 'wilcox.test',
                        leg = 'CAF_3',ylab = 'GSVA Score')
fig2g
ggsave('results/Fig2f.pdf',fig2g,height = 5,width = 9)

#C4
tumor.pathway.score.group5=tumor.score.copy[tumor.score.copy$seurat_clusters=='CAF_4',]
fig2h<-Muti_Boxplot(dat
=tumor.pathway.score.group5[,as.character(unique(tumor.pathway$OG.TSG))],
                        group = tumor.pathway.score.group5$copykat.pred,
                        group_cols = ggsci::pal_lancet()(9)[c(2,1)],
                        test_method = 'wilcox.test',
                        leg = 'CAF_4',ylab = 'GSVA Score')
```

```
fig2h
ggsave('results/Fig2g.pdf',fig2h,height = 5,width = 9)
save.image('all.RData')
```
