# Supplementary figures and images for: Identification macrophage signatures in prostate cancer by single-cell sequencing and machine learning
Source: Cancer Immunol Immunother. 2024 Feb 13;73(3):41. doi: 10.1007/s00262-024-03633-5 (PMC10864475; doi:10.1007/s00262-024-03633-5)

**A**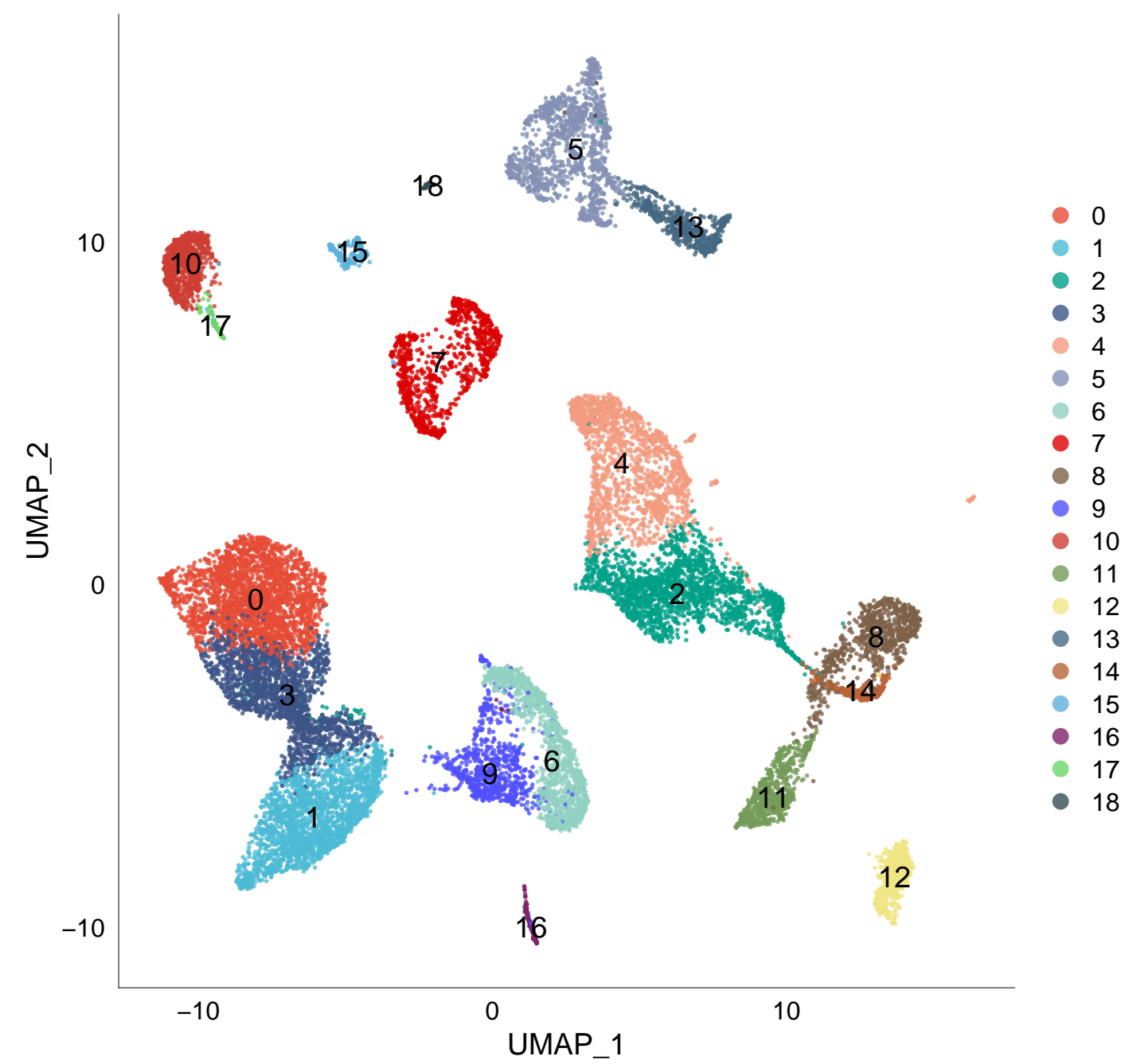**B**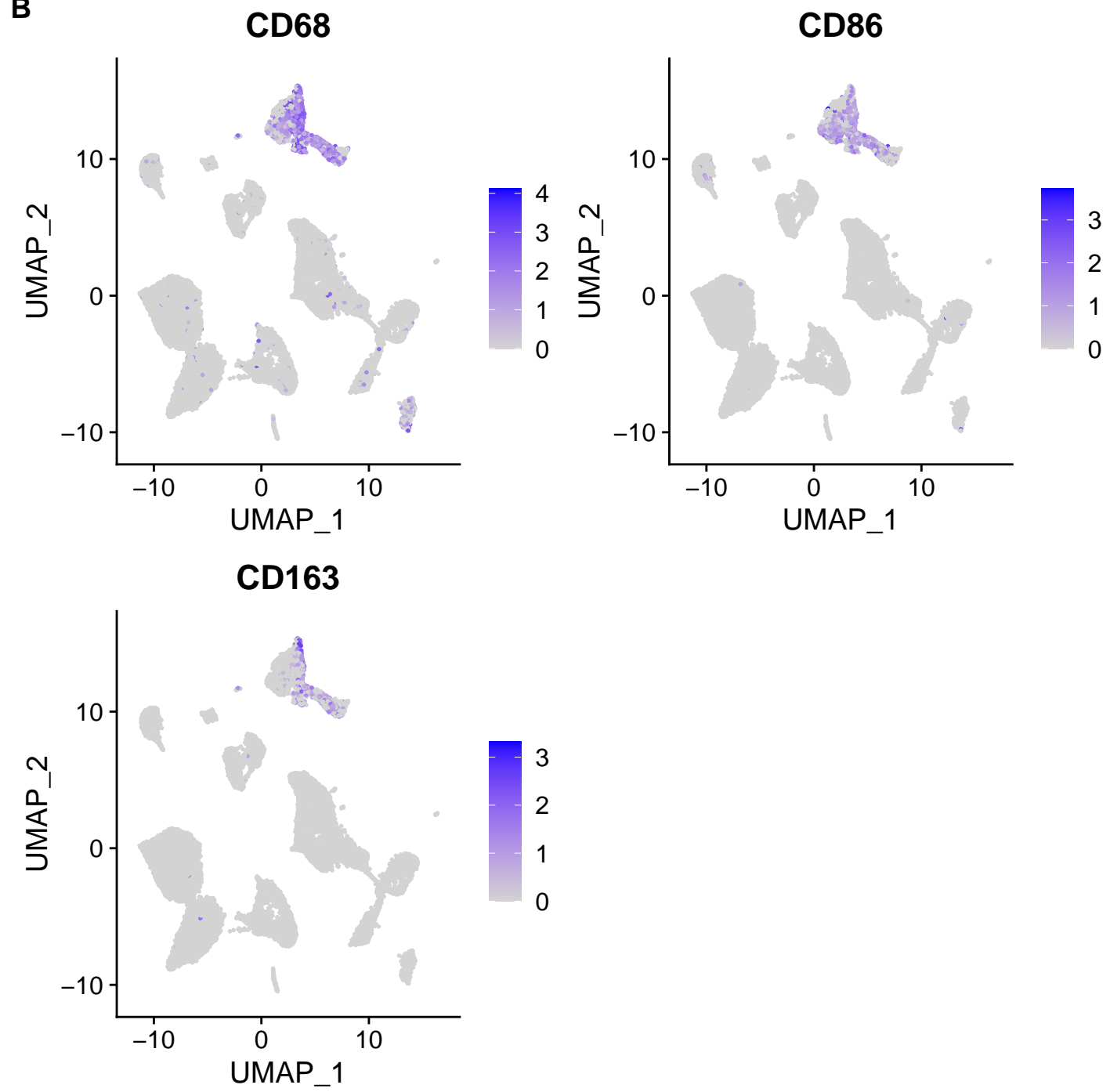**C**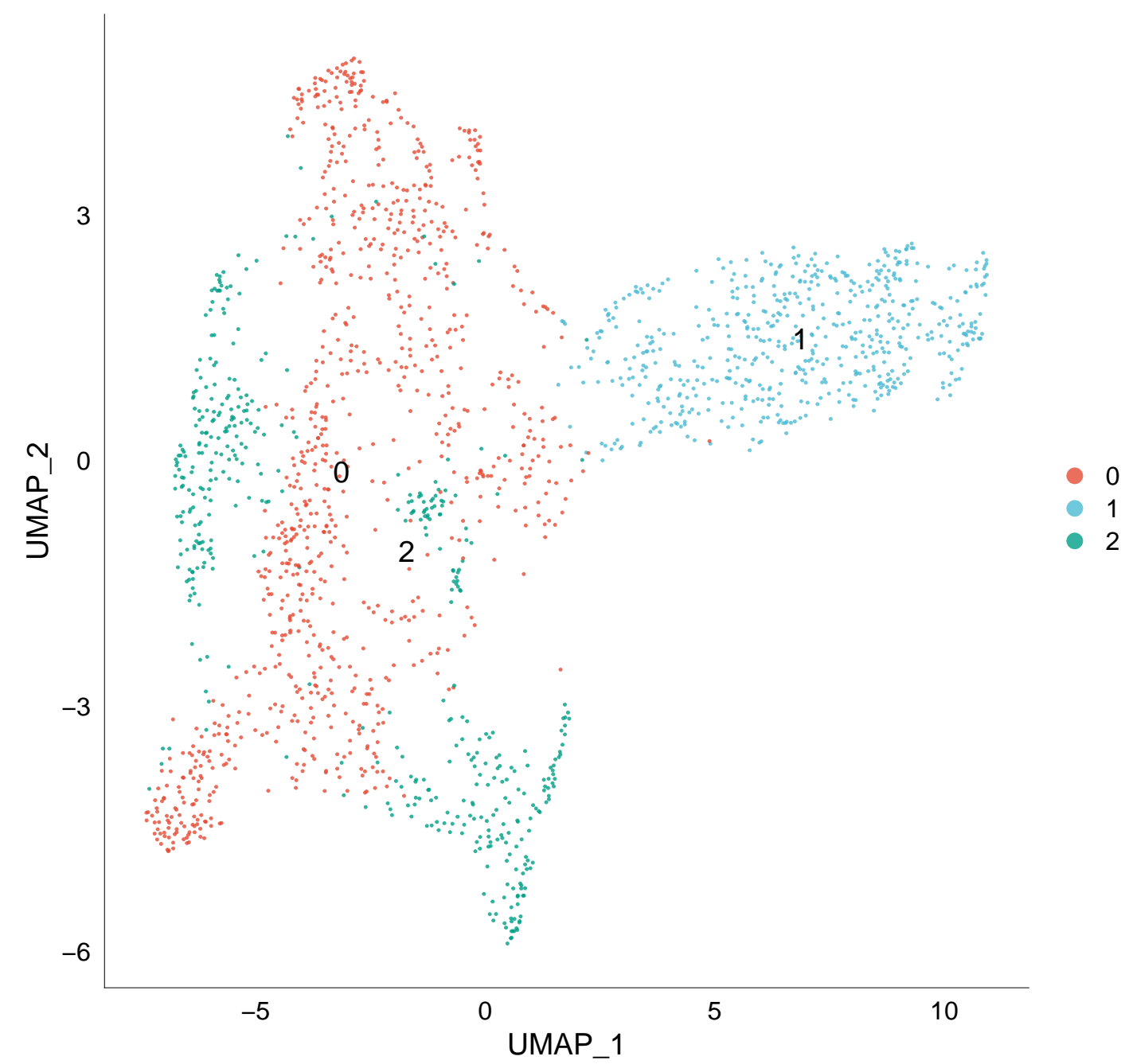**D**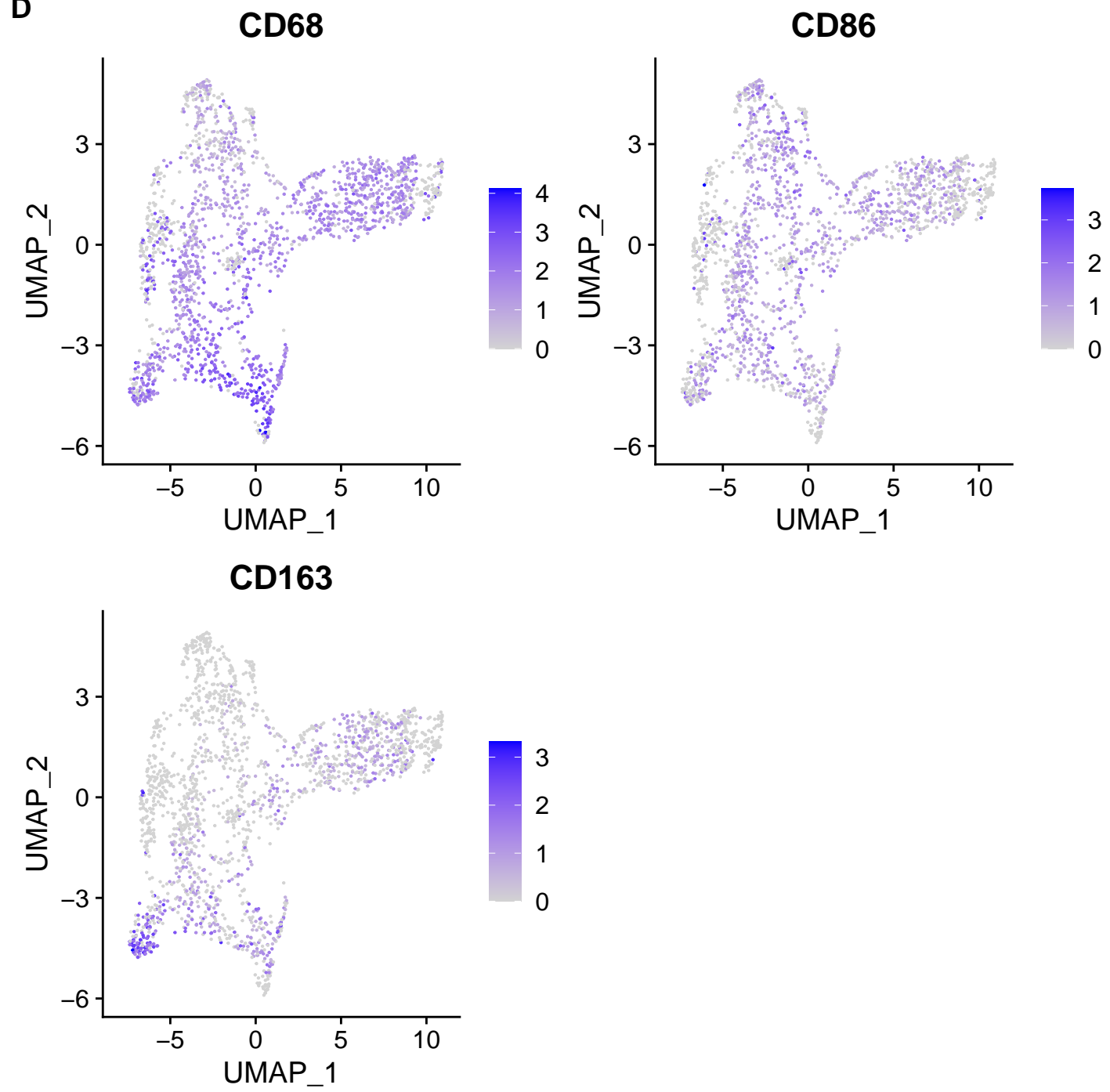

Supplement: Supplementary file 2 — Supplementary file2 (PDF 4706 kb) [file 262_2024_3633_MOESM2_ESM.pdf]

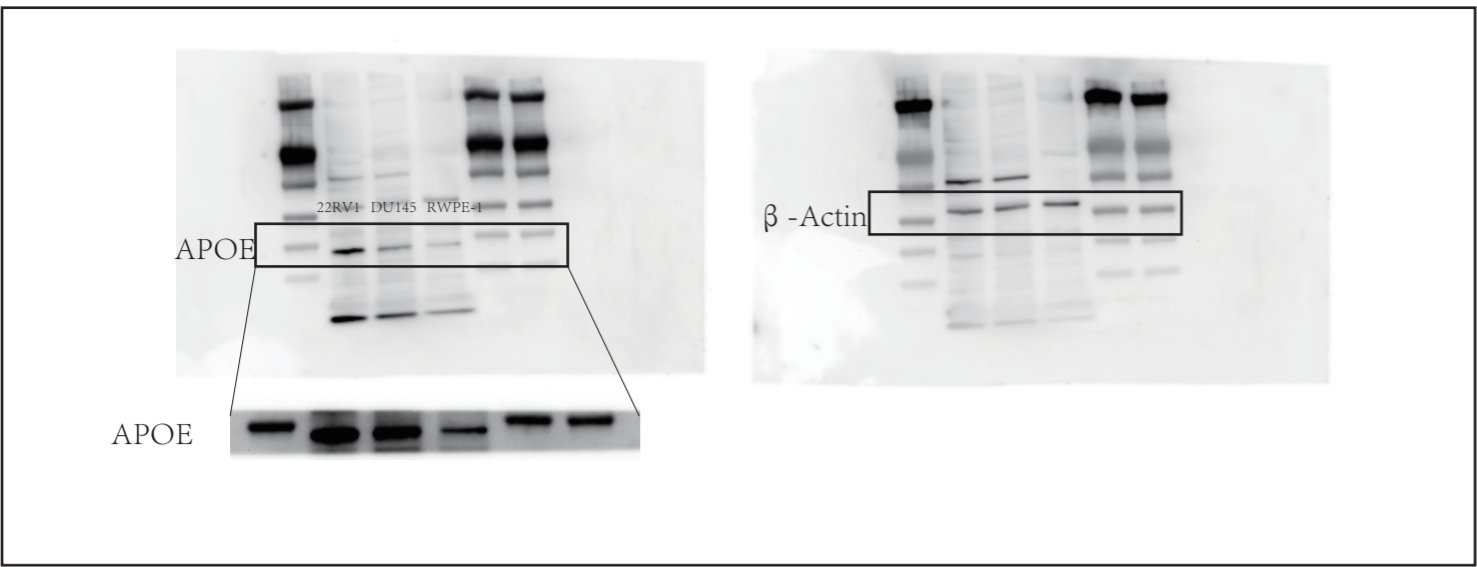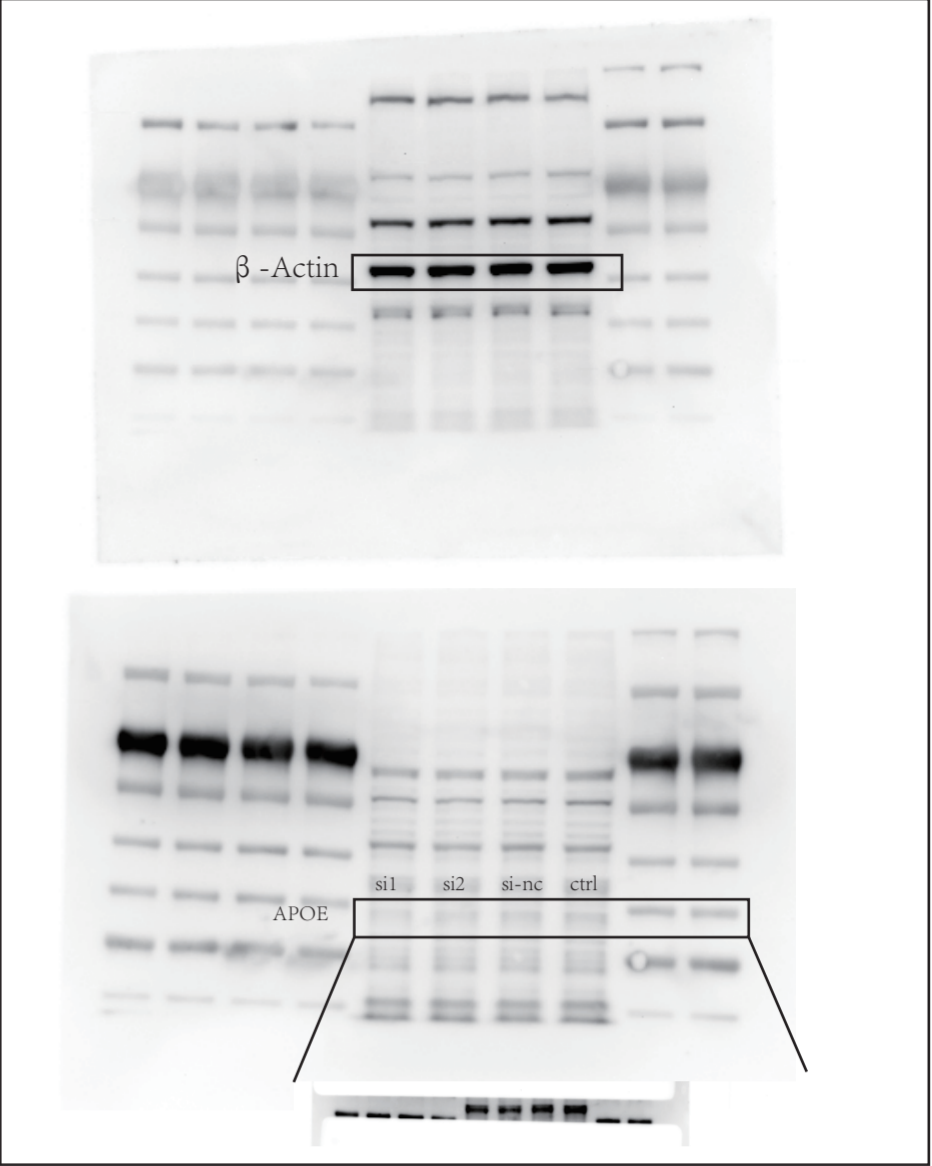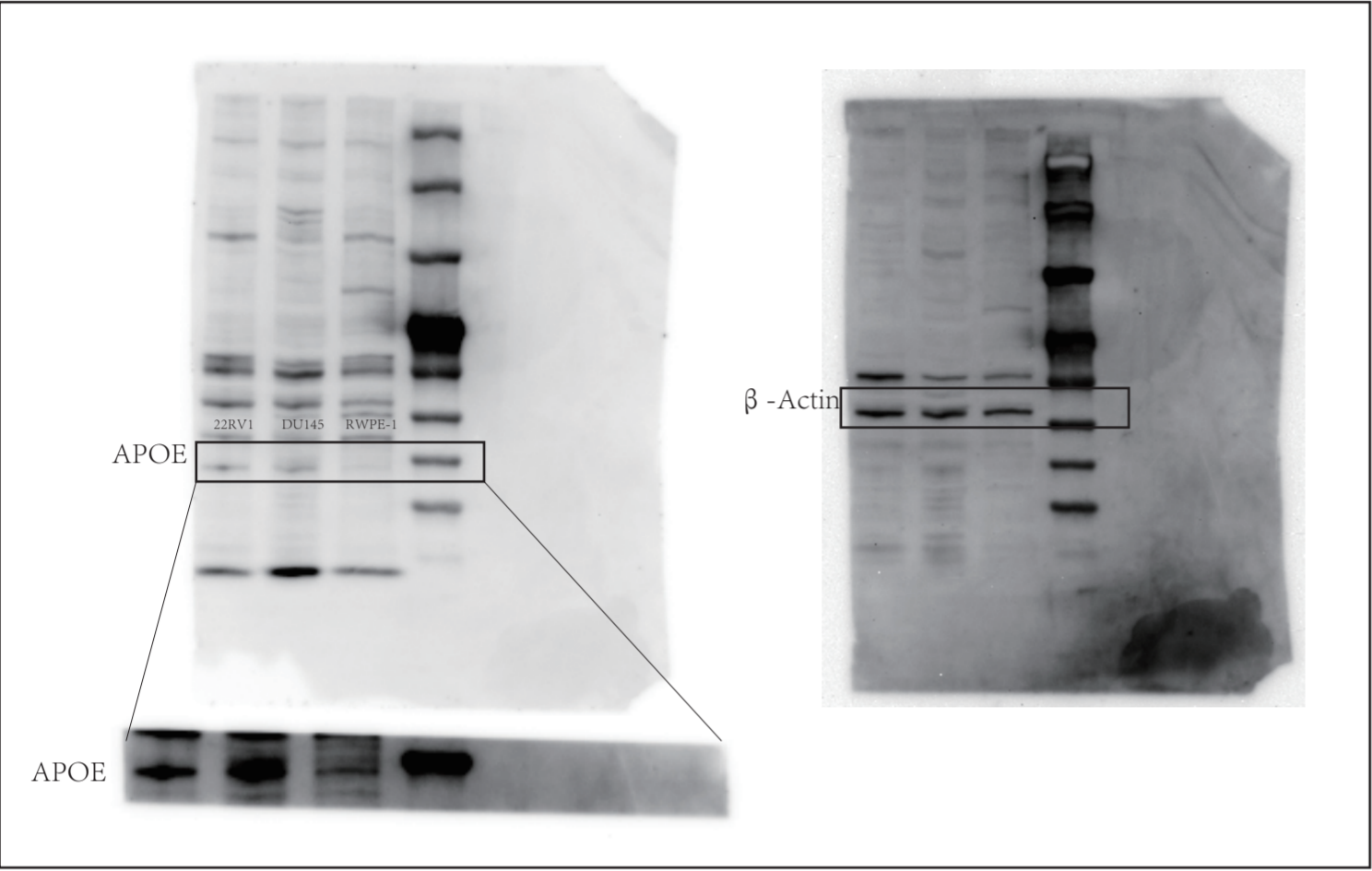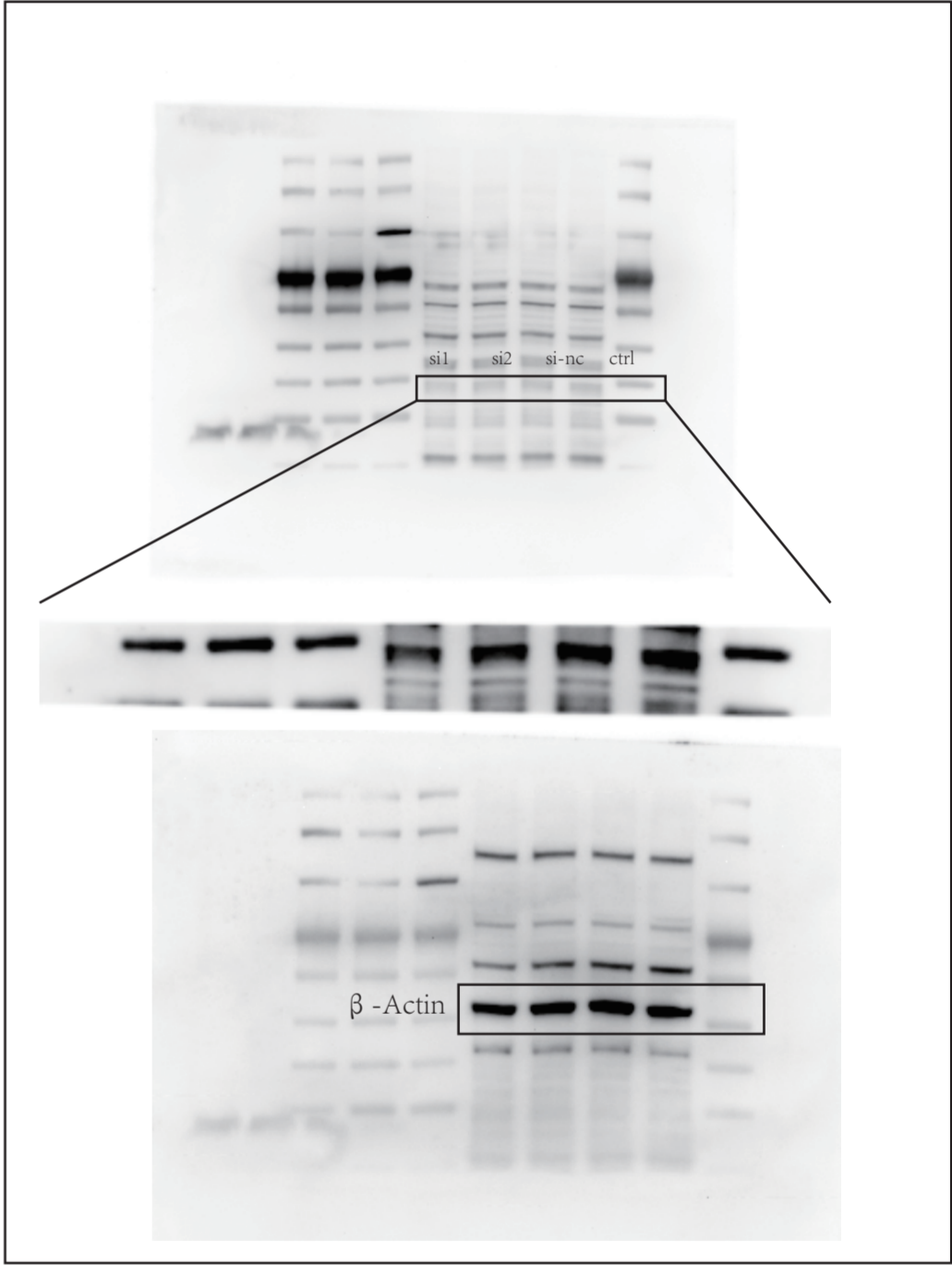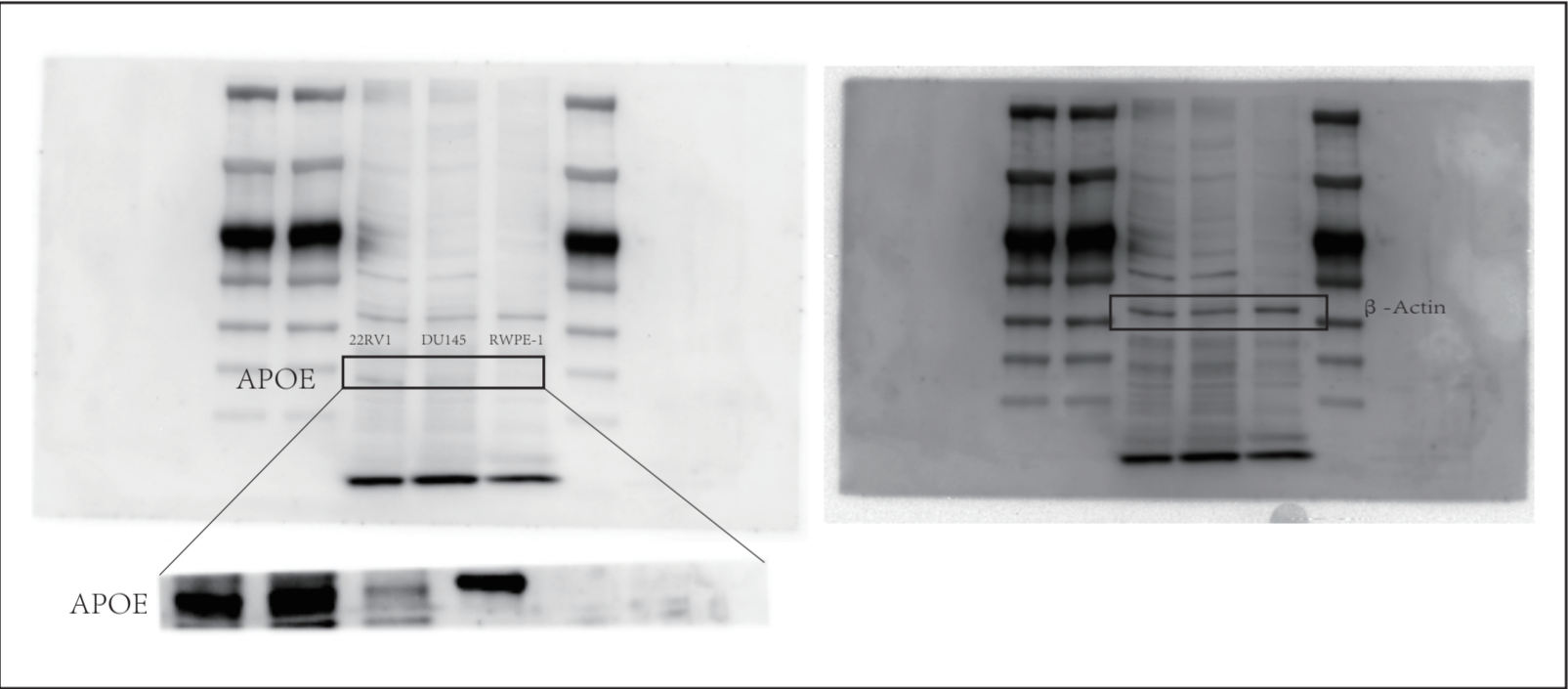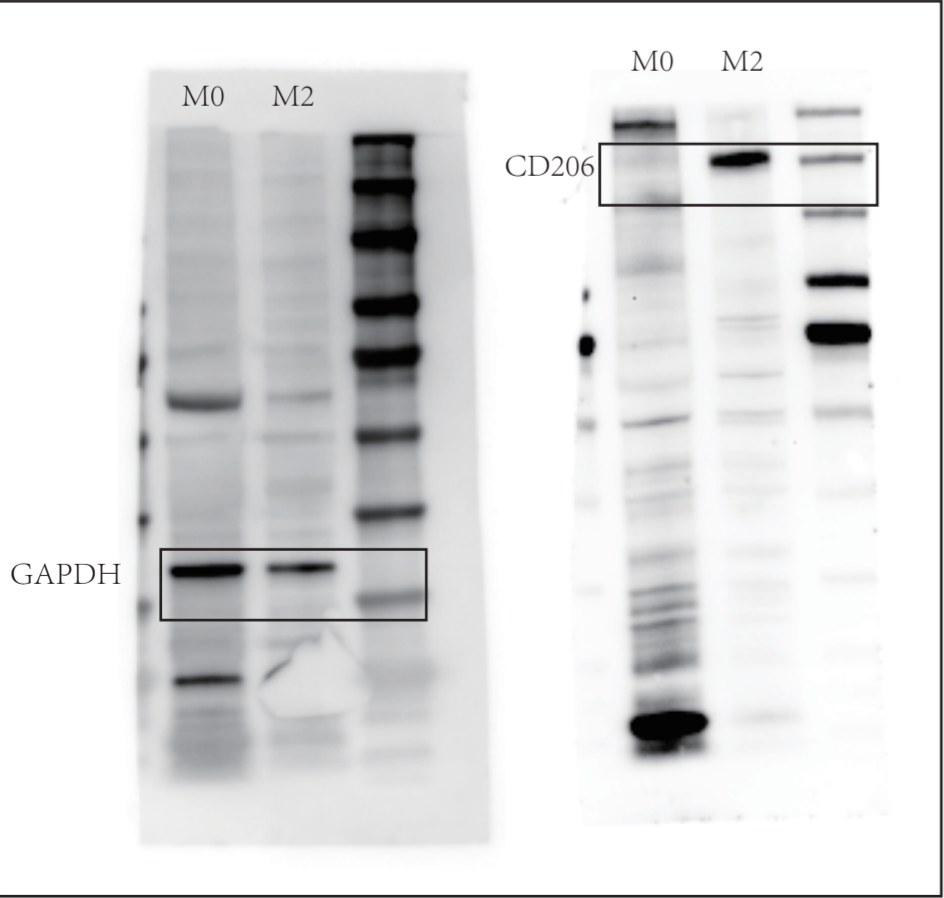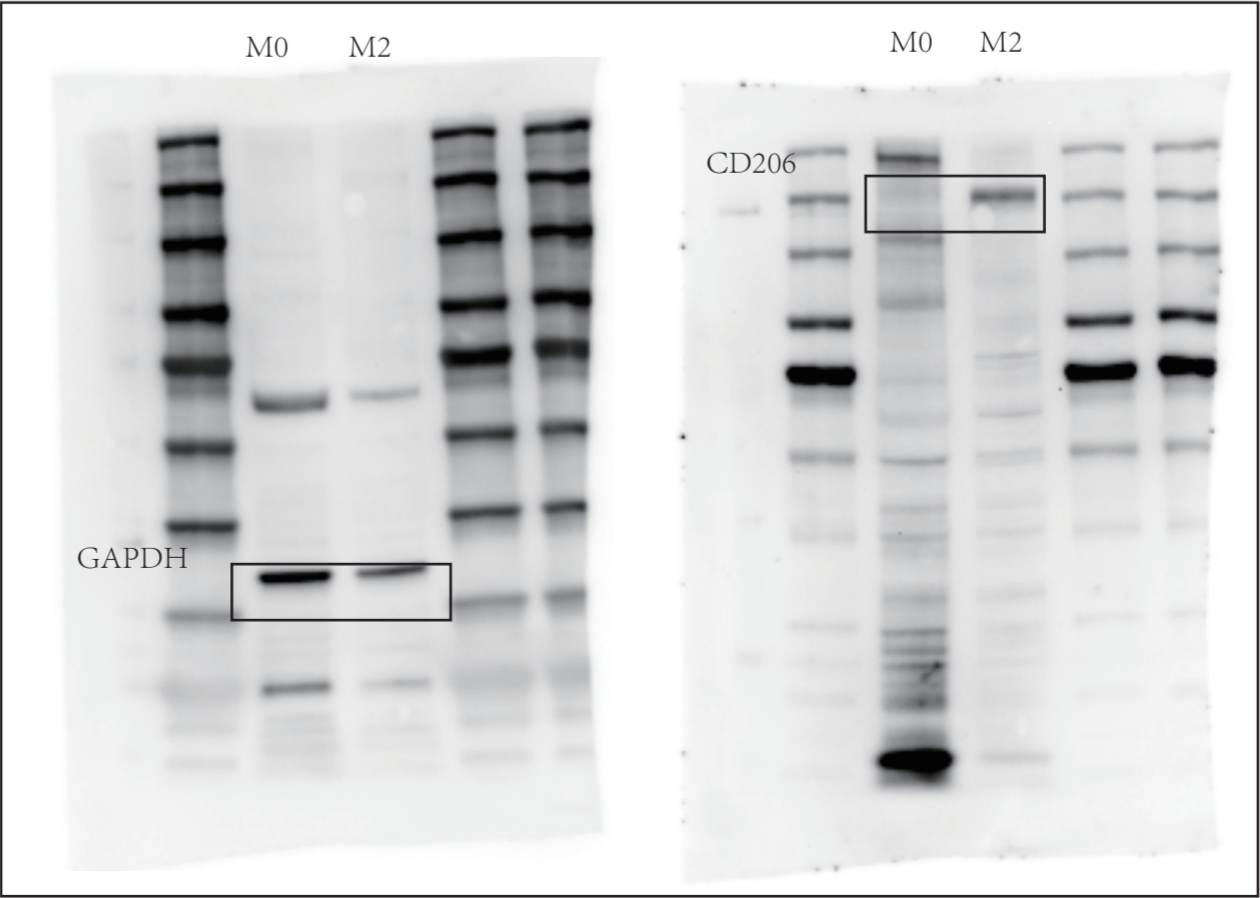

Supplement: Supplementary file 3 — Supplementary file3 (PDF 2127 kb) [file 262_2024_3633_MOESM3_ESM.pdf]
